# Celline: a flexible tool for one-step retrieval and integrative analysis of public single-cell RNA sequencing data

**DOI:** 10.3389/fbinf.2025.1684227

**Published:** 2025-12-11

**Authors:** Yuya Sato, Toru Asahi, Kosuke Kataoka

**Affiliations:** 1 Graduate School of Advanced Science and Engineering, Waseda University, Tokyo, Japan; 2 Comprehensive Research Organization, Waseda University, Tokyo, Japan; 3 Institute of Engineering, Tokyo University of Agriculture and Technology, Tokyo, Japan

**Keywords:** single-cell RNA-seq, public databases, pipeline, integration, python, data management

## Abstract

Single-cell RNA sequencing (scRNA-seq) has generated a rapidly expanding collection of public datasets that provide insight into development, disease, and therapy. However, researchers lack an end-to-end solution for seamlessly retrieving, preprocessing, integrating, and analyzing these data because existing tools address only isolated steps and require manual curation of accessions, metadata, and technical variability, known as batch effects. In this study, we developed Celline, a Python package that executes an entire workflow using a single-line commands per step. Celline automatically gathers raw single-cell RNA-seq data from multiple public repositories and extracts metadata using large language models. It then wraps established tools, including Scrublet for doublet removal, Seurat and Scanpy for quality control and cell-type annotation, Harmony and scVI for batch correction, and Slingshot for trajectory inference, into one-line commands, enabling seamless integrative analyses. To validate Celline-acquired data quality and the integrated framework’s practical utility, we applied it to 2 mouse brain cortex datasets from embryonic days 14.5 and 18. Technical validation demonstrated that Celline successfully retrieved data, standardized metadata, and enabled standard analyses that removed low-quality cells, annotated 11 major cell types, improved integration quality (scIB score +0.22), and completed trajectory analysis. Thus, Celline transforms scattered public scRNA-seq resources into unified, analysis-ready datasets with minimal effort. Its modular design allows pipeline extension, encourages community-driven advances, and accelerates the discovery of single-cell data.

## 1 Introduction

Single-cell RNA sequencing (scRNA-seq) enables transcriptomic profiling at single-cell resolution, facilitating studies on disease mechanisms, cellular composition, cell type discovery, developmental lineage reconstruction, tumor heterogeneity, immune profiling, and drug discovery applications (Van de Sande et al., 2023; Chang et al., 2024). The rapid evolution of the scRNA-seq technology has reduced costs and broadened its adoption, leading to an extensive accumulation of publicly available scRNA-seq datasets (Arya et al., 2025). A search for “scRNA-seq” currently returns 78,655 samples in the GEO (Gene Expression Omnibus) database, illustrating this exponential growth.

Despite this wealth of data, methods for systematic reuse remain immature, complicating the unified retrieval, preprocessing, integration, and downstream analysis of publicly archived scRNA-seq data (Wang et al., 2025; Wu and Tang, 2025). Current analytical solutions typically only address isolated steps, leaving researchers without comprehensive workflows for end-to-end analyses. The R package GEfetch2R enables data collection from public databases, but requires manual management of dataset identifiers and lacks automated metadata retrieval across multiple repositories (Song et al., 2023). Curated databases, such as DISCO, provide only selected subsets, which prevents the analysis of user-selected datasets (Li et al., 2022). Recent tools such as scExtract and scBaseCount automate specific aspects of data retrieval, but they focus on literature-based approaches or single repositories, lacking multi-repository integration with automated metadata standardization (Wu and Tang, 2025; Youngblut et al., 2025).

Additionally, inconsistent quality control across studies complicates data integration. When researchers apply different QC parameters or thresholds, even using the same tools, the resulting variability can obscure biological signals. This standardization is particularly important given the inherent variability in sample quality due to differences in tissue processing and RNA extraction methods across laboratories.

These limitations are compounded by the significant heterogeneity across repositories. Differences exist among public databases, such as Gene Expression Omnibus (GEO), Sequence Read Archive (SRA), China National Center for Bioinformation (CNCB), and ArrayExpress, in terms of data formats (FASTQ, BAM), data submission standards, and batch effects, making comprehensive analysis challenging (Rustici et al., 2021).

To overcome these obstacles, we developed Celline, a Python package that executes typical workflows using a single command. Celline manages diverse public scRNA-seq data, extracts metadata using large language models, and wraps established tools for data acquisition, preprocessing, cell-type annotation, batch correction, and trajectory inference. Critically, inconsistent quality control parameters and diverse tools across studies hinder reproducible analyses of public scRNA-seq data. Celline addresses this by wrapping established tools into a unified workflow, enabling researchers to apply consistent filtering criteria across datasets. Similarly, unified cell-type annotation remains challenging due to varying annotation standards. Celline provides a standardized framework that facilitates consistent cell-type identification across public datasets. Users can easily perform customized downstream analyses, as Celline integrates seamlessly with existing environments. It supports direct invocation of tools such as Python, R, and others, all within a modular, extensible framework. Celline supports execution on cluster servers and scales efficiently for extensive data collection, allowing researchers to utilize the full range of public scRNA-seq resources with minimal effort and improved reproducibility.

To clarify Celline’s unique position among existing tools, Table 1 compares its key features with representative scRNA-seq workflow tools.

**TABLE 1:** Comparison of Celline with existing scRNA-seq tools across key functional categories.

Tool	Primary focus	Multi-repo accession resolution	Metadata standardization	Automated data download	Downstream tool integration	Platform	References
Celline	Multi-repository automated retrieval and LLM-driven standardization	✓ (GEO, ArrayExpress, CNCB, etc.)	✓ (LLM-powered)	✓	✓ (Multiple methods)	Python CLI	This study
scExtract	LLM-automated annotation from published literature	✗ (Literature-based)	✓ (Article PDF)	Partial	✓ (Scanorama, CellHint)	Python	Wu and Tang (2025)
scBaseCount	SRA-focused automated processing	✓ (SRA only)	✓ (AI agent)	✓	✓	Python	bioRxiv 2025
DISCO	Curated tissue atlas integration platform	✗ (Pre-curated)	✗ (Manual curation)	✓ (Pre-processed)	✓ (Atlas-level)	Web/R/Python	Li et al. (2022)
GEfetch2R	Basic GEO data downloading	Partial (GEO only)	✗	✓	✗	R	Song et al. (2023)

Celline uniquely integrates automated multi-repository retrieval (GEO, SRA, etc.), LLM-based metadata standardization, and both preprocessing and downstream analysis in a unified command-line workflow.

## 2 Methods

### 2.1 Overview of celline

Celline is a Python library that can be installed with a single command using “pip install celline.” A comprehensive tutorial is available at https://kataoka-k-lab.github.io/CellineDocs/.

Celline can be executed from the command-line interface or Python code. This study explains the command-line workflow, as it provides the most straightforward use. Users can extend functionality by creating custom classes that inherit the “CellineFunction” base class. Celline automatically detects function classes, allowing users to call them with the command “celline run” followed by the function name, thereby extending the toolkit without altering the core code. Example commands are provided in Supplementary Table S2.

**FIGURE 1:**
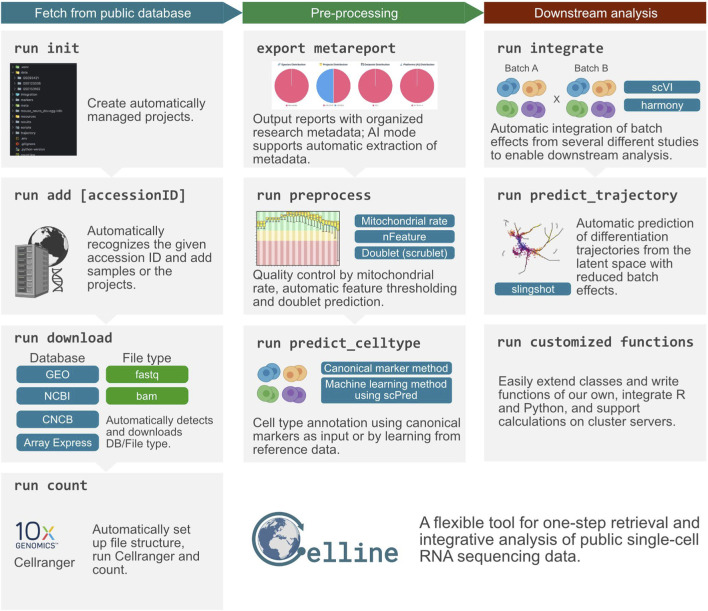
Overview of the Celline workflow for public scRNA-seq data. The pipeline was divided into three color-coded stages. (Left, teal) Fetch from public database: “run init” creates a project structure; “run add [accessionID]” registers studies by accession; “run download” automatically detects the source database and retrieves raw FASTQ/BAM files; and “run count” organizes the directory tree and executes Cell Ranger to generate gene-barcode matrices. (Center, green) Pre-processing: “export metareport” compiles and visualizes study metadata; “run preprocess” performs quality control (mitochondrial-read filtering, feature-count thresholds, doublet removal with Scrublet); and “run predict_celltype” assigns cell identities either by canonical markers or an scPred-based machine-learning approach. (Right, brown) Downstream analysis: “run integrate” removes inter-study batch effects via scVI or Harmony; “run predict_trajectory” reconstructs differentiation lineages with Slingshot in the corrected latent space; and “run customized functions” allows users to extend the framework with bespoke R/Python modules or cluster-server jobs. Collectively, Celline offers a flexible, one-step solution for rapid retrieval, comprehensive processing, and integrative analysis of public single-cell RNA sequencing datasets.

To accommodate evolving tool versions, Celline implements an extensible architecture. Users can create custom functions via “celline create <MyFunction>” command, enabling adaptation to new tool versions without modifying core code. These custom functions inherit from the CellineFunction base class and are automatically detected by the framework, allowing seamless integration of version-specific implementations. Currently, job scheduling relies on Torque (PBS) systems. An overview of Celline’s standard workflow is shown in Figure 1.

Celline implements custom exception handling (NullPointException, RSessionNotFoundException, RException) with console logging via Python’s logging module. Dependency management uses “pyproject.toml” for version pinning, and “mise” for managing development tools including “uv”. Project configuration and version information are stored in “setting.toml”, while sample metadata is managed through “metadata.xlsx” within each project directory.

### 2.2 Add accession to the project

The add function integrates open-access scRNA-seq datasets into a project. Users issue a “celline run add” command followed by an accession identifier. Celline recognizes the identifier type, resolves related projects and sample accessions, and collects detailed metadata. When a GSM identifier from the GEO database is provided, the program locates the parent GSE project accession and the associated SRA run identifiers to download the linked data. During this process, Celline crawls the repository pages, determines whether each file is available in FASTQ, BAM, or any other format, and records the results in the project directory.

Beyond public accession identifiers, Celline also accommodates directly obtained data from various sources. Users can integrate private FASTQ files, BAM files, or preprocessed count matrices into the Celline workflow by executing “celline run add <CustomID>”, where CustomID is a user-defined identifier. This command initiates an interactive session that guides users through data registration, allowing them to specify file paths, data types, and associated metadata. This functionality is particularly valuable for researchers working with proprietary datasets, clinical samples, or data obtained through direct collaboration. Once added, these custom datasets seamlessly integrate with all downstream Celline functions—including preprocessing, integration, and trajectory analysis—ensuring that both public and private data can be analyzed within the same unified framework. The metadata for custom datasets can be manually edited and incorporated into the project’s metareport, maintaining consistency across all data sources.

Users can summarize the metadata collected by running the “celline export metareport” command. Adding the “--ai” option engages a large language model based on OpenAI GPT, which extracts and standardizes unstructured information, such as tissue names and sequencing platforms, to produce a normalized report ready for meta-analysis.

### 2.3 Automatically downloading and counting 10x scRNA-seq data

The download function leverages the metadata obtained through the addition process to automatically prepare the sequence data for analysis. It organizes files into a directory structure compatible with the specified counting tool. If the data are provided as BAM files, Celline automatically converts them to FASTQ format using appropriate conversion utilities. A key feature of Celline is the automated management of these processes through basic jobs configured based on user-defined thread settings in the configuration file. Celline monitors the status of each job by sequentially managing downloads and counting tasks to ensure efficient operations. This automated job management system enhances scalability, facilitating large-scale analysis of archived scRNA-seq datasets on cluster servers.

For the counting process, Celline supports multiple alignment and quantification tools to accommodate diverse sequencing protocols. By default, it uses Cell Ranger’s “count” command for 10x Genomics data, leveraging its optimized algorithms for droplet-based sequencing. However, recognizing that many public datasets utilize different protocols, Celline also integrates STARsolo, which users can specify via “celline run count--tool starsolo” (Kaminow et al., 2021). This option enables processing of Smart-seq2, Drop-seq, and other droplet-based or plate-based protocols. This alternative option allows processing of various sequencing protocols not supported by Cell Ranger, including Smart-seq2, Drop-seq, and other plate-based or droplet-based methods.

### 2.4 Preprocessing scRNA-seq data

The preprocess function, invoked with “celline run preprocess,” applies three common quality control steps. First, cells with a mitochondrial gene fraction greater than five percent are removed, as high mitochondrial content often indicates stressed or dying cells (Luecken and Theis, 2019). Second, Celline filters cells based on nFeatures, defined as the number of unique genes detected in a cell. Thresholds are determined automatically by calculating the median absolute deviation of nFeatures across all cells to filter outliers that fall below or above three times the median absolute deviation from the median. This typically excludes cells with extremely low or excessively high gene counts that may represent empty droplets or multiplets. Third, potential doublets are identified and excluded using the Scrublet package (Wolock et al., 2019). All quality control metrics are visualized in diagnostic plots, and the pipeline sets a flag to True only for cells that passed through every filter. Downstream analyses reference this flag to ensure that only high-quality cells contribute to the results. The automated workflow produces consistent and explainable quality standards across datasets, enabling reliable integration and comparison.

### 2.5 Automated cell-type annotation

The manual annotation of cell types in diverse samples is often error-prone, subjective, and time-consuming, particularly for large datasets. To address these challenges, Celline provides the “celline run predict_celltype” command, offering two distinct annotation strategies. The first approach utilizes canonical marker genes that are validated and are robust cell-type-specific markers based on existing research. Users define marker sets along with adjustable weights indicating marker reliability (e.g., 1.0 for highly reliable markers, 0.8 for less established markers) and directions (positive or negative expression) to improve annotation precision. For example, excitatory neurons are typically annotated as positive for Slc17a6 and negative for Gad1 and Gad2, thereby enhancing the annotation robustness.

The second strategy employs supervised machine learning using scPred, which requires a well-annotated reference dataset for training (Alquicira-Hernandez et al., 2019). Users can either utilize pre-built reference models or create custom references tailored to their specific tissue or experimental context. To build a custom reference, users execute “celline run buildcelltypereference” with their annotated scRNA-seq data, which trains an scPred model capturing cell-type-specific expression patterns. This command accepts various input formats including AnnData objects from cell atlases or previously annotated in-house datasets. The trained reference model is then applied to new samples via “celline run predict_celltype--method scpred--reference <model_path>”. While this reference-based approach generally provides robust predictions, accuracy may decrease for samples that differ significantly from the training data or in complex samples such as organoids where standardized cell lineage annotations are not yet fully established.

### 2.6 Integration and batch correction of multiple datasets

To fully utilize publicly available data, it is essential to combine datasets from multiple studies into a unified dataset for downstream analyses. However, integrating datasets from different studies introduces technical variations known as batch effects, which can distort gene expression profiles (Ryu et al., 2023). These effects may result from differences in experimental conditions such as temperature and humidity, reagent kit versions, or sequencing depth. Several methods have been developed to correct the batch effects. Harmony is a graph-based method widely recognized for its high batch correction capability (Korsunsky et al., 2019). However, it only operates in the PCA space, making it unsuitable for downstream analyses requiring a corrected full-gene expression matrix. Therefore, variational autoencoder (VAE)-based methods, including scVI, are commonly used because they generate corrected gene expression values for downstream analyses (Lopez et al., 2018). Celline provides the “celline run integrate” command, offering workflows for both Harmony-based and scVI-based integration. When Harmony is selected, Celline computes the PCA, applies harmony correction to the PCA coordinates, and generates neighbor graphs and UMAP embeddings. If the scVI is chosen, Celline trains an scVI model using sample IDs as batch keys. After model training, Celline generates latent space embeddings from which neighbor graphs and UMAP are derived. Downstream analyses are performed using predicted normalized expression values obtained from the trained scVI model.

### 2.7 Trajectory inference for differentiation analysis

Predicting differentiation trajectories is essential for developmental studies. Celline provides the “celline run predict_trajectory” command, which automates trajectory inference using Slingshot (Street et al., 2018). The command loads the integrated scVI model produced by “celline run integrate,” predicts latent embeddings, and calls Slingshot to construct a minimum spanning tree and assign pseudotime. To accurately reconstruct differentiation trajectories, selecting an appropriate root cluster is critical. In Celline, users are required to explicitly specify the root cluster using the “--root-cluster” parameter based on their biological knowledge of the system under study. This manual specification ensures that trajectory inference aligns with established developmental biology principles specific to each tissue and experimental context.

As optional decision support, Celline provides visualization of cluster-level metrics that may aid in root selection. These include cell cycle scores calculated from the S phase and G2/M phase genes listed in the Seurat “cc.genes.updated.2019” dataset. Users can visualize these scores through heatmaps and UMAP overlays, which helps identify proliferative clusters that often correspond to progenitor populations. However, these metrics serve only as Supplementary Material, and the final root cluster selection remains the user’s responsibility based on their domain expertise.

Slingshot uses this root to infer lineages in latent space. Celline saves the inferred pseudotime table, lineage-to-cell-type mapping, and graphical outputs, which include UMAP overlays, heat maps of cluster scores, and minimum spanning tree plots colored by clusters, cell types, and marker gene expression. These outputs support a detailed inspection of developmental paths and can be used directly in subsequent analyses.

## 3 Results

As a species-agnostic data preparation tool, Celline’s performance is independent of biological context such as species or disease models. To demonstrate its functionality, we analyzed 2 mouse brain developmental datasets representing different embryonic stages. We retrieved embryonic day 14.5 (E14.5) and embryonic day 18 (E18) C57BL/6 brain samples, sequenced using the 10x Chromium platform, from two separate studies: GSE153162 by Di Bella et al. and GSE93421 by Zheng et al. (2017), Di Bella et al. (2021) (Table 2). Note, the E18 sample (GSM2453043) represents a pooled collection from cortex, hippocampus, and subventricular zone, encompassing multiple neurogenic regions. This multi-region composition provides a comprehensive view of late embryonic brain cell types, though it precludes region-specific analyses. The following results demonstrate technical validation of Celline’s data acquisition framework, not novel biological discoveries.

**TABLE 2:** Summary of datasets used in this study.

Mus musculus					
GEO datasets ID	Study	Dev. age [embryonic]	Tissue	Sequencer	URL
GSE153162	Di Bella et al. (2021)	E14.5	Cortex	Illumina HiSeq 2000	https://www.ncbi.nlm.nih.gov/geo/query/acc.cgi?acc=GSE153162
GSE93421	Zheng et al. (2017)	E18	Whole Brain (cortex, hippocampus, and subventricular zone were pooled)	Illumina HiSeq 4000	https://www.ncbi.nlm.nih.gov/geo/query/acc.cgi?acc=GSE93421

Homo sapiens				
GEO datasets ID	Study	Tissue	Sequencer	URL
GSE115189	Freytag et al., F1000Res. (2018)	Peripheral blood mononuclear cells (PBMCs)	Illumina HiSeq 2500	https://www.ncbi.nlm.nih.gov/geo/query/acc.cgi?acc=GSE115189

GEO, Datasets ID: identifier of the NCBI, gene expression omnibus database.

### 3.1 Technical validation of automated data acquisition and preprocessing

**FIGURE 2:**
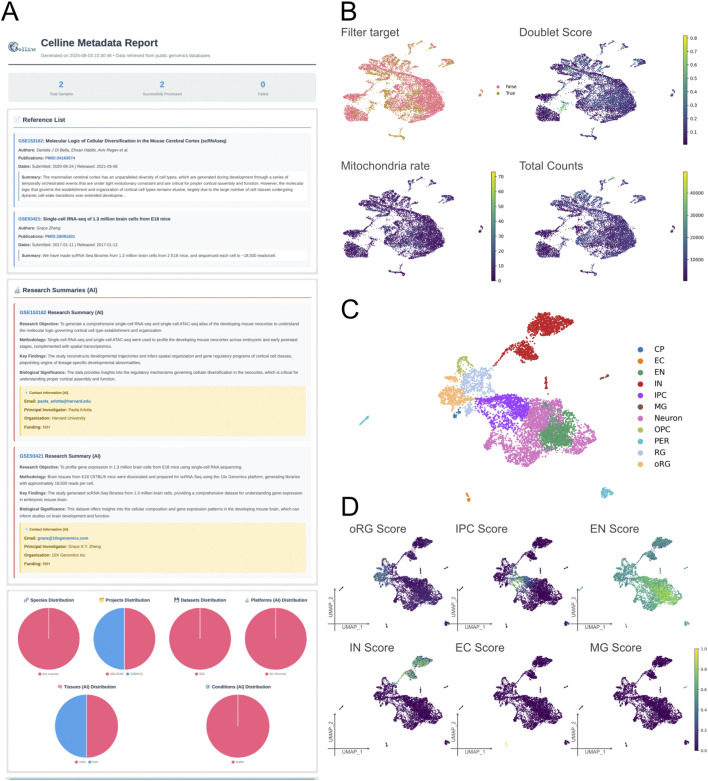
Technical validation of representative outputs generated by Celline for a mouse cortical-development case study (E18 sample only). **(A)** Metadata report (Meta report). A fully formatted HTML report will be generated via the command “celline export metareport”, listing the imported references, summarizing key study attributes (objectives, methodologies, sample sizes, key findings, funding, and contact e-mails), and visualizing global metadata distributions (species, projects, diseases, platforms, tissues, and conditions) as pie charts. **(B)** Quality Control Diagnostics. Four UMAP overlays illustrate the key QC metrics used in the run preprocessing step: cells flagged for removal (filter target), doublet score (Scrublet), mitochondrial-read percentage, and total UMI counts. These views help users interactively fine-tune filtering thresholds. **(C)** Cell-type annotation. The UMAP shows 11 predicted populations—CP (choroid plexus), EC (endothelial cell), EN (excitatory neuron), IN (inhibitory neuron), IPC (intermediate progenitor cell), MG (microglia), Neuron (immature neuron), OPC (oligodendrocyte progenitor cell), oRG (outer radial glia), PER (pericyte), and RG (radial glia)—assigned by the run predict_celltype module. **(D)** Gene-signature scoring. The UMAP heat maps display module scores for five representative signatures (IPC, EN, IN, EC, and MG), illustrating how run predict_celltype complements discrete labels with continuous, cell-type-specific activation gradients. Together, these panels demonstrate the end-to-end capability of Celline for metadata curation, rigorous QC, integrative clustering, and rich technical validation of the pipeline of public single-cell RNA-Seq datasets.

The samples were added to a single project by executing “celline run add” with accession identifiers GSM4635075 and GSM2453043. We then generated a metadata report with “celline export metareport--ai” (Figure 2A). The crawler extracted species and citation information from GEO webpages, and the large language model engine-predicted sequencing platform, organ, and condition fields, confirming that the metadata were consistent across the two samples (Figure 2A).

LLM-based metadata extraction was validated against manually curated data from 96 samples (638 fields across 10 categories). Overall accuracy: 90.8% exact-match, 97.0% meaning-match. Protocol and sequencer fields achieved 100% accuracy; treatment and genotype showed lower accuracy (80.0%, 78.4%) due to description complexity. In less accurate extractions, there were cases where Celline annotated a sample as C57BL/6J, while manual curation data annotated it as wildtype. These discrepancies stemmed from differences in the referenced sources or the comparison targets, and to the best of our knowledge, they were not due to hallucinations (Supplementary Table S3).

To evaluate Celline’s data acquisition capabilities, we assessed computational time and scalability across three core workflows: accession resolution, LLM-based metadata extraction, and data download. Performance benchmarks showed that accession resolution time ranged from 6.9s to 39.2s depending on dataset complexity and sample count, with GEO datasets exhibiting log-linear scaling (R^2^ = 0.995) that remained under 40s even for projects containing ∼200 samples. LLM metadata extraction averaged 5–7s per sample with minimal dependency on text size (R^2^ = 0.064), demonstrating consistent performance across varying metadata complexity. Data download time scaled with file size and network bandwidth (Supplementary Figure S4). Downstream processing (counting, quality control, cell-type annotation, integration) depends on wrapped tools’ performance; refer to original publications for benchmarks.

Next, we conducted quality control using “celline run preprocess,” which estimated doublet scores, calculated mitochondrial gene fractions, and determined optimal nFeature thresholds for each cell (Figure 2B; Supplementary Figures S1A–S1D). For this quality control demonstration and subsequent cell-type annotation (Sections 3.1, 3.2), we focused on the later developmental stage sample, GSM2453043 (E18), which showed a diversified cellular composition. Note that the integration and trajectory analyses presented in later sections (Sections 3.3, 3.4) utilize both E14.5 and E18 samples combined. The median absolute deviation analysis set an optimal nFeature range of 536–3,446 genes per cell (Supplementary Figure S1A) and a total UMI upper limit of 9,654 counts (Supplementary Figure S1B). The mitochondrial filter retained a default five percent threshold (Supplementary Figure S1C). The Scrublet tool predicted a doublet score threshold of 0.366 (Supplementary Figure S1D). Most of the retained cells fell within narrow ranges of total and gene counts (Supplementary Figure S1E) and showed moderate mitochondrial content (Supplementary Figure S1F). Seventy percent of the cells passed all filters; among the discarded cells, high mitochondrial content was the most frequent exclusion factor (Supplementary Figures S1G, S1H). Therefore, the pipeline applied consistent quality control across the dataset. Users may make these filters less stringent by adjusting the median absolute deviation multiplier or mitochondrial threshold, if desired. Overall, a single command produces stable quality control metrics and clear visualizations for downstream analysis.

### 3.2 Performance evaluation of automated cell-type annotation

Canonical marker-based cell-type annotation was performed using the established weighted marker gene sets provided in Supplementary Table S1 (Figure 2C). The E18 sample’s multi-region composition (cortex, hippocampus, and subventricular zone) contributed to the diverse cell types identified. Only CP, EC, EN, IN, IPC, MG, Neuron, OPC, PER, oRG and RG cell types were annotated successfully (Figure 2C; Supplementary Figure S2A). These annotated cell types formed distinct clusters consistent with their biological lineages. Neuronal cell types clustered closely, whereas endothelial cells, pericytes, and microglia formed separate clusters (Figure 2C). Annotation accuracy was validated by computing expression-based scores for each cell type using defined canonical markers. The scores aligned well with the annotation outcomes, confirming the accuracy and reliability of the annotation (Figure 2D; Supplementary Figure S2B). Overall, our canonical marker-based approach accurately annotated the cell types. The reference-based cell-type annotation implemented via scPred was not evaluated in this study, as it directly utilized the existing scPred method and was therefore considered outside the scope of our validation.

### 3.3 Integration of datasets from different studies

**FIGURE 3:**
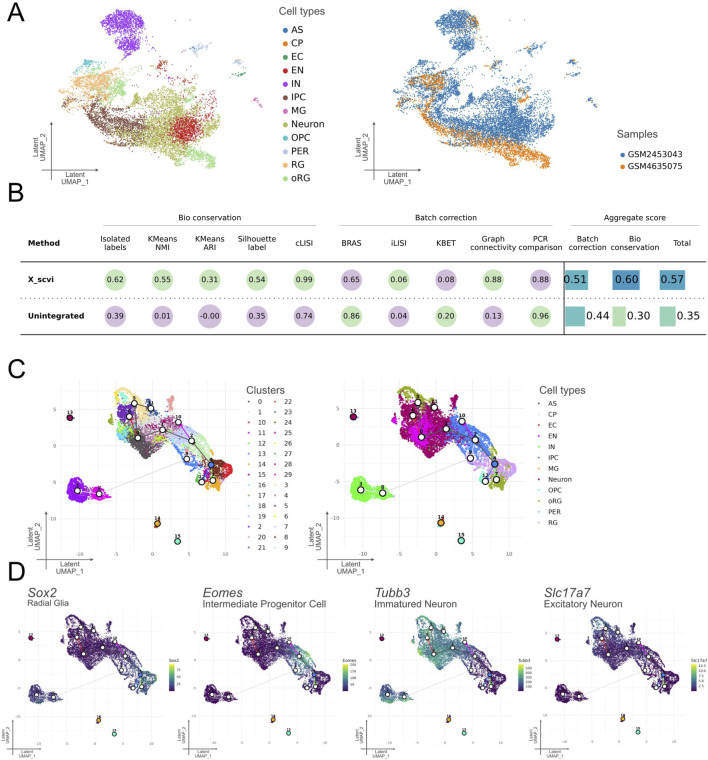
Technical assessment of integration quality, clustering, and trajectory inference for two embryonic mouse-cortex datasets (Integrated E14.5 + E18). **(A)** scVI-integrated latent space. Left: UMAP colored according to the 11 predicted cell-type classes: CP, EC, EN, IN, IPC, MG, Neuron, OPC, oRG, PER and RG. Right: The same embedding is colored by the two original samples, illustrating successful batch mixing. **(B)** Quantitative assessment of integration. Bioconservation metrics (isolated label score, k-means NMI/ARI, Silhouette label, and cLISI) and batch-correction metrics (BRAS, iLISI, kBET, graph connectivity, and PCR) were reported for the scVI-corrected data (X_scvi) versus unintegrated concatenation. Aggregate scores showed higher batch correction (0.51) and biological conservation (0.60) for scVI, yielding a superior overall score (0.57) compared with the raw merge (0.35). **(C)** Clustering versus annotation of the trajectory lineages. Left: Leiden clusters (hulls and numeric labels) identified in the integrated space; Right: correspondence of these clusters to the predicted cell-type classes in **(A)**, confirming good biological coherence. The trajectory divergence is consistent with current knowledge, indicating that a robust trajectory lineage can be predicted. **(D)** Marker-gene validation. Expression heatmaps of the UMAP for four canonical markers, *Sox2* (radial glia), *Eomes* (intermediate progenitor cells), *Tubb3* (immature neurons), and *Slc17a6* (excitatory neurons), demonstrated specific enrichment in their expected regions, supporting the accuracy of the automated cell-type assignments. Taken together, the expression levels of the predicted cell type marker genes were consistent with the neural differentiation trajectory.

In this section, we demonstrate Celline’s integration capabilities using both developmental timepoints—E14.5 (GSM4635075) and E18 (GSM2453043)—which were preprocessed and annotated independently in the previous sections. Datasets from different studies, preprocessed and annotated automatically, were integrated using the scVI mode in the “celline run integrate” command. To enable downstream analysis, scVI was used with the accession IDs of the samples as batch keys, generating a unified UMAP visualization (Figure 3A). The two batches showed successful integration, with cells from identical cell types clustered closely together despite originating from different studies and being annotated independently (Figure 3A). Quantitative evaluation of batch integration using the scib-metrics package revealed improved scores for both “Batch correction” and “Bio conservation” metrics after correction, with an overall increase of 0.22 in total score (Figure 3B) (Büttner et al., 2019). Collectively, the integration process effectively reduces batch effects, allowing the combined dataset to be analyzed as a unified resource.

### 3.4 Validation of trajectory analysis workflow integration

Using the integrated E14.5 and E18 dataset from the previous section, we performed trajectory inference to reconstruct developmental lineages spanning both timepoints. Using the “celline run predict_trajectory” command, we performed integrated trajectory inference on latent embeddings obtained from datasets combined via “celline run integrate.” Cells were grouped into 30 distinct clusters (clusters 0–29), and differentiation trajectories were inferred using Slingshot, called internally within Celline (Figure 3C). Trajectory mapping revealed clear differentiation paths from the RG toward glial lineages, specifically OPC, and toward neuronal lineages via IPC. The neuronal lineage further branches into separate pathways leading to IN and EN neurons. Importantly, cell types derived from the yolk sac or mesodermal origin, such as MG, EC, and PER, were correctly excluded from the RG-based trajectories, indicating consistency with expected patterns and technically demonstrates end-to-end execution (Figure 3C). The expression patterns of key marker genes, such as *Sox2* for RG, *Eomes* for IPC, *Tubb3* for Neurons, and *Slc17a6* for EN, aligned well with the expected cell types and differentiation stages, supporting the technical validity of the batch-corrected gene expression metrics produced by the scVI model (Figure 3D). To determine the root cluster, we examined the cell cycle scores across clusters and manually selected the root cluster based on these scores (Supplementary Figure S3A).

### 3.5 Species-agnostic design

Celline’s architecture operates on standardized sequencing formats and metadata structures rather than species-specific features. The workflow—accession retrieval, LLM-based metadata standardization, and unified analysis—functions equivalently across organisms without requiring species-specific modifications. This design enables analysis of human, mouse, or other species data through identical command structures. To validate this capability, we applied the identical workflow to human PBMC scRNA-seq data, confirming equivalent functionality across species (Supplementary Figure S5).

## 4 Discussions

Celline represents a powerful and effective tool for managing, collecting, preprocessing, and analyzing the rapidly expanding collection of scRNA-seq data available in public databases. As the number of publicly accessible scRNA-seq datasets continues to grow, they have become an essential platform for effective utilization of these resources. Celline offers a robust and reproducible analysis workflow through a simple single-line command interface, a feature that, to our knowledge, integrates multi-repository retrieval, LLM-based normalization, and unified downstream analysis into a single end-to-end workflow.

Another notable advantage of Celline is its extensibility. Users can readily create, implement, and improve additional functions, thus facilitating the development and sharing of advanced analytical workflows. Community-driven extensibility is vital for fostering collaboration and innovation within the single-cell analysis community, significantly enhancing the reproducibility and transparency of analytical methods.

The practical demonstration provided by our analyses using Celline illustrates its effectiveness in integrating datasets from different studies. This integration capability enables researchers to seamlessly obtain, unify, and analyze diverse datasets from public repositories, thereby streamlining complex analytical pipelines. Such capabilities are particularly valuable for large-scale meta-analyses that require integration across multiple studies and conditions, as demonstrated in recent studies analyzing immune responses across cancer types and comparing vascularization strategies in cerebral organoids (Sato et al., 2023; Shorer et al., 2025). We have successfully applied Celline in continuous aging analysis studies, where it facilitated efficient retrieval and standardization of multiple datasets.

Furthermore, Celline offers enhanced flexibility in handling diverse data scenarios. Users can seamlessly integrate their own datasets into the workflow, enabling the incorporation of custom or specialized data alongside publicly available resources. However, some limitations remain, such as the lack of a user-friendly web-based interface and native support for the integration of scATAC-seq or spatial transcriptomic data. Addressing these challenges through future enhancements is critical for maximizing the utility of ever-expanding public datasets. For spatial transcriptomics, we plan to integrate frameworks like Squidpy and SpatialData through Celline’s extensible architecture, preserving spatial coordinates during retrieval. Similarly, scATAC-seq support could be added via tools such as ArchR or Signac.

Additionally, Celline currently uses different computational libraries for various analyses (e.g., Scanpy for integration visualization and Slingshot for trajectory analysis), which can result in slight variations in UMAP projections even when analyzing the same dataset. While this does not affect the biological conclusions, achieving perfect visual consistency across different library implementations remains a technical challenge. Future versions may standardize visualization tools to ensure uniform representations across all analyses.

## 5 Limitations and future work

The tool lacks a graphical user interface and requires command-line proficiency. Support is currently limited to scRNA-seq data, with native support for scATAC-seq, spatial transcriptomics, and CITE-seq absent in the current version. Different wrapped tools may produce slight visualization variations due to implementation differences, though biological conclusions remain consistent. While our validation with 96 manually curated samples demonstrated 90.8% exact-match accuracy (97.0% meaning-match accuracy), the LLM-based metadata extraction may produce hallucinations or misannotations for other samples, particularly in complex experimental conditions. While major repositories (GEO, SRA, ArrayExpress, CNCB) are supported, some regional or specialized databases remain unsupported.

### 5.1 Future development

For spatial transcriptomics, we plan to integrate frameworks like Squidpy and SpatialData through Celline's extensible architecture. Similarly, scATAC-seq support could be added via tools such as ArchR or Signac.

## Data Availability

Celline (v1.1.0, RRID: SCR_027272) is an open-source package released under an MIT license. The source code is available at https://github.com/Kataoka-K-Lab/Celline. The exact versions used in this study are permanently archived in Zenodo (https://doi.org/10.5281/zenodo.17274556), along with all analysis scripts required to reproduce the results. Comprehensive user documentation is provided at https://kataoka-k-lab.github.io/CellineDocs. All the calculations were performed using CentOS Linux release 7.4.1708 and Ubuntu 22.04.2.

## Supplementary material

SUPPLEMENTARY FIGURE 1 Quality-control threshold selection and filtering summary. **(A)** Distribution of detected genes per cell (“gene complexity”). Vertical dashed lines mark the lower (536 genes) and upper (3,446 genes) cut-offs, and the median (1,991 genes) is shown as a reference. **(B)** Distribution of total UMI counts per cell. The red dashed line indicates the upper threshold (9,654 counts), with a median of 4,771. **(C)** Distribution of mitochondria-derived transcript percentages. Cells above the 5.0 % threshold (red dashed line) are flagged for removal (median: 3.0 %). **(D)** Histogram of the Scrublet doublet scores. A score of 0.366 (vertical dashed line) is used to exclude the putative doublets (median = 0.071). **(E)** Scatter plot of total counts versus gene complexity. Blue points denote cells passing all QC criteria, and red points are filtered out by at least one rule. **(F)** Scatter plot of total counts versus mitochondrial percentage with the same pass/fail color as in (E). **(G)** Bar chart summarizing the absolute number and proportion of cells removed by each criterion (gene complexity, total counts, mitochondrial percentage, and doublets) and the combined total (30 % of all cells). **(H)** Pie chart showing the overall outcome: 8,130 cells (70 %) are retained and 3,483 cells (30 %) were discarded after QC. These panels illustrate how Celline transparently visualizes threshold choices and their impact on dataset quality.

SUPPLEMENTARY FIGURE 2 Cell-type composition and signature-score landscape of the annotated embryonic cortex dataset. **(A)** Global composition. Left: Pie chart showing the proportional abundance of the 11 predicted cell classes—EN (excitatory neuron, 36.5%), IN (inhibitory neuron, 18.1%), Neuron (13.1%), IPC (intermediate progenitor cell, 11.6%), oRG (outer radial glia, 7.8%), RG (radial glia, 6.4%), and minor populations PER (pericyte, 1.7%), OPC (oligodendrocyte precursor, 1.5%), MG (microglia, 0.9%), EC (endothelial, 0.4%) and CP (choroid plexus, 0.2%)—summarizing their relative contributions in GSE93421 dataset. Right: Corresponding bar chart displaying absolute cell counts for each class, highlighting the predominance of excitatory neurons and the balanced representation of neuronal, progenitor and glial lineages. **(B)** Module-score heatmaps. UMAP overlays of gene-signature scores for each cell type (oRG, RG, IPC, OPC, AS, EN, IN, Neuron, PER, MG, CP) reveal spatial enrichment patterns that corroborate discrete annotations in Figure 2C–D. Scores are calculated by Celline’s “run predict_celltype” module using curated marker sets, providing a continuous measure of cell-identity strength across the manifold.

SUPPLEMENTARY FIGURE 3 Pseudotime-derived functional scores across the integrated embryonic cortex manifold. **(A)** Cell-cycle score. The combined S- and G2/M-phase module scores revealed a confined zone of cycling progenitors and low proliferation in differentiated populations.

SUPPLEMENTARY FIGURE 4 Scalability and performance benchmarks of Celline's data acquisition workflows. **(A)** Accession resolution performance across four representative datasets (GSE70580, GSE87069, GSE93374, E-MTAB-5061), showing resolution times ranging from 6.9s to 39.2s depending on dataset complexity and repository structure. **(B)** LLM metadata extraction performance distribution across all tested datasets, with median extraction times of 5-7s per sample and interquartile ranges indicating consistent performance across diverse metadata formats. **(C)** Relationship between metadata text size and LLM processing time, demonstrating minimal correlation (R^2^=0.064) and indicating that extraction performance remains stable regardless of text complexity. **(D)** Scalability analysis of accession resolution time versus sample count in GEO datasets, revealing log-linear scaling (R^2^=0.995) that remains under 40s even for large projects containing ∼200 samples. Downstream processing (counting, quality control, cell-type annotation, integration) depends on wrapped tools' performance; refer to original publications for benchmarks.

SUPPLEMENTARY FIGURE 5 Species-agnostic validation with human scRNA-seq data. Demonstration of Celline's species-agnostic capability using human PBMC scRNA-seq data. The identical workflow—accession retrieval, LLM-based metadata extraction and quality control—was applied to human data without species-specific modifications, confirming architectural independence from organism-specific features. Results show successful processing with quality metrics comparable to mouse data analysis.

SUPPLEMENTARY TABLE 1 Annotation markers. File for annotations used in annotation (“celline run predict_celltype”). Enter the target organ. In this study, the format is suitable for the mouse brain.

SUPPLEMENTARY TABLE 2 Example Celline commands. Common command examples for Celline workflows including basic project initialization, data download, preprocessing, cell-type annotation, integration, and trajectory inference. Examples demonstrate single-sample, multi-sample, and HPC cluster execution modes.

SUPPLEMENTARY TABLE 3 LLM metadata extraction accuracy. Validation of LLM-based metadata extraction against manually curated data from 96 single-cell RNA-seq samples. Overall exact-match accuracy: 90.8%, meaning-match accuracy: 97.0%. Protocol and sequencer fields achieved 100% accuracy. Lower accuracy was observed for sex (40.0%), treatment (80.0%), and genotype (78.4%) fields.

## Abbreviations

RG, Radial glia; oRG, Outer radial glia; IPC, Intermediate progenitor cell; EN, Excitatory neuron; IN, Inhibitory neuron; CP, Choroid plexus; SPN, Subplate neuron; OPC, Oligodendrocyte precursor cell; OL, Oligodendrocyte; EC, Endothelial cell; PER, Pericyte; SMC, Smooth muscle cell; FIB, Fibroblast; EPEN, Ependymal cell; MG, Microglia.

## References

[B1] Alquicira-Hernandez J. Sathe A. Ji H. P. Nguyen Q. Powell J. E. (2019). scPred: accurate supervised method for cell-type classification from single-cell RNA-seq data. Genome Biol. 20, 264. 10.1186/s13059-019-1862-5 31829268 PMC6907144

[B2] Arya A. Tripathi P. Dubey N. Aier I. Kumar Varadwaj P. (2025). Navigating single-cell RNA-sequencing: protocols, tools, databases, and applications. Genomics Inf. 23, 13. 10.1186/s44342-025-00044-5 40382658 PMC12085826

[B3] Büttner M. Miao Z. Wolf F. A. Teichmann S. A. Theis F. J. (2019). A test metric for assessing single-cell RNA-seq batch correction. Nat. Methods 16, 43–49. 10.1038/s41592-018-0254-1 30573817

[B4] Chang X. Zheng Y. Xu K. (2024). Single-cell RNA sequencing: technological progress and biomedical application in cancer research. Mol. Biotechnol. 66, 1497–1519. 10.1007/s12033-023-00777-0 37322261 PMC11217094

[B5] Di Bella D. J. Habibi E. Stickels R. R. Scalia G. Brown J. Yadollahpour P. (2021). Molecular logic of cellular diversification in the mouse cerebral cortex. Nature 595, 554–559. 10.1038/s41586-021-03670-5 34163074 PMC9006333

[B6] Kaminow B. Yunusov D. Dobin A. (2021). STARsolo: accurate, fast and versatile mapping/quantification of single-cell and single-nucleus RNA-seq data. bioRxiv. 10.1101/2021.05.05.442755

[B7] Korsunsky I. Millard N. Fan J. Slowikowski K. Zhang F. Wei K. (2019). Fast, sensitive and accurate integration of single-cell data with Harmony. Nat. Methods 16, 1289–1296. 10.1038/s41592-019-0619-0 31740819 PMC6884693

[B8] Li M. Zhang X. Ang K. S. Ling J. Sethi R. Lee N. Y. S. (2022). DISCO: a database of deeply Integrated human single-Cell omics data. Nucleic Acids Res. 50, D596–D602. 10.1093/nar/gkab1020 34791375 PMC8728243

[B9] Lopez R. Regier J. Cole M. B. Jordan M. I. Yosef N. (2018). Deep generative modeling for single-cell transcriptomics. Nat. Methods 15, 1053–1058. 10.1038/s41592-018-0229-2 30504886 PMC6289068

[B10] Luecken M. D. Theis F. J. (2019). Current best practices in single-cell RNA-Seq analysis: a tutorial. Mol. Syst. Biol. 15, e8746. 10.15252/msb.20188746 31217225 PMC6582955

[B11] Rustici G. Williams E. Barzine M. Brazma A. Bumgarner R. Chierici M. (2021). Transcriptomics data availability and reusability in the transition from microarray to next-generation sequencing. bioRxiv. 10.1101/2020.12.31.425022

[B12] Ryu Y. Han G. H. Jung E. Hwang D. (2023). Integration of single-cell RNA-seq datasets: a review of computational methods. Mol. Cells 46, 106–119. 10.14348/molcells.2023.0009 36859475 PMC9982060

[B13] Sato Y. Asahi T. Kataoka K. (2023). Integrative single-cell RNA-seq analysis of vascularized cerebral organoids. BMC Biol. 21, 245. 10.1186/s12915-023-01711-1 37940920 PMC10634128

[B14] Shorer O. Pinhasi A. Yizhak K. (2025). Single-cell meta-analysis of T cells reveals clonal dynamics of response to checkpoint immunotherapy. Cell Genom 5, 100842. 10.1016/j.xgen.2025.100842 40187353 PMC12143341

[B15] Song Y. Wang J. Gao J. (2023). *GEfetch2R*: fetching single-cell/bulk RNA-seq data from public repositories to R and benchmarking the subsequent format conversion tools. bioRxiv. 10.1101/2023.11.18.567507 PMC13147242 41964930

[B16] Street K. Risso D. Fletcher R. B. Das D. Ngai J. Yosef N. (2018). Slingshot: cell lineage and pseudotime inference for single-cell transcriptomics. BMC Genomics 19, 477. 10.1186/s12864-018-4772-0 29914354 PMC6007078

[B17] Van de Sande B. Lee J. S. Mutasa-Gottgens E. Naughton B. Bacon W. Manning J. (2023). Applications of single-cell RNA sequencing in drug discovery and development. Nat. Rev. Drug Discov. 22, 496–520. 10.1038/s41573-023-00688-4 37117846 PMC10141847

[B18] Wang P. Liu W. Wang J. Liu Y. Li P. Xu P. (2025). ScCompass: an integrated multi-species scRNA-seq database for AI-ready. Adv. Sci. (Weinh.) 12, e2500870. 10.1002/advs.202500870 40317650 PMC12224968

[B19] Wolock S. L. Lopez R. Klein A. M. (2019). Scrublet: computational identification of cell doublets in Single-cell transcriptomic data. Cell Syst. 8, 281–291.e9. 10.1016/j.cels.2018.11.005 30954476 PMC6625319

[B20] Wu Y. Tang F. (2025). scExtract: leveraging large language models for fully automated single-cell RNA-seq data annotation and prior-informed multi-dataset integration. Genome Biol. 26, 174. 10.1186/s13059-025-03639-x 40537825 PMC12178070

[B21] Youngblut N. D. Carpenter C. Nayebnazar A. Adduri A. Shah R. Ricci-Tam C. (2025). scBaseCount: an AI agent-curated, uniformly processed, and autonomously updated single cell data repository. bioRxiv. 10.1101/2025.02.27.640494 42753696

[B22] Zheng G. X. Y. Terry J. M. Belgrader P. Ryvkin P. Bent Z. W. Wilson R. (2017). Massively parallel digital transcriptional profiling of single cells. Nat. Commun. 8, 14049. 10.1038/ncomms14049 28091601 PMC5241818

