# Supplementary material for: Celline: a flexible tool for one-step retrieval and integrative analysis of public single-cell RNA sequencing data
Source: Front Bioinform. 2025 Dec 11;5:1684227. doi: 10.3389/fbinf.2025.1684227 (PMC12738925; doi:10.3389/fbinf.2025.1684227)
Supplement: Supplementary file 1 [file DataSheet2.pdf]

LLM Metadata Extraction Accuracy Summary
(96 samples, 638 evaluable fields)

Field	Exact Match	Meaning Match	Evaluable	Total Samples
protocol	100.0%	100.0%	96/96	96
sequencer	100.0%	100.0%	96/96	96
tissue	88.5%	100.0%	96/96	96
cell_type	95.7%	100.0%	46/96	96
organism	83.3%	100.0%	96/96	96
age	78.4%	88.2%	51/96	96
sex	40.0%	80.0%	5/96	96
treatment	80.0%	80.0%	5/96	96
genotype	78.4%	78.4%	51/96	96
experiment_type	95.8%	100.0%	96/96	96
**OVERALL**	**90.8%**	**97.0%**	**638**	**96**

GSM1811163 (Series: GSE70580)
Evaluable: 6 fields | Exact: 5 | Meaning: 1 | Mismatch: 0

Field	Manual/Ground Truth	LLM Extraction	Match
protocol	Smart-seq2	Smart-seq2	✓ Exact
sequencer	Illumina HiSeq 2000	Illumina HiSeq 2000	✓ Exact
tissue	tonsil	tonsil	✓ Exact
cell_type	innate lymphoid cells	Innate lymphoid cells	✓ Exact
organism	human	Homo sapiens	≈ Meaning
experiment_type	single-cell RNA-seq	single-cell RNA-seq	✓ Exact

GSM1811162 (Series: GSE70580)
Evaluable: 6 fields | Exact: 5 | Meaning: 1 | Mismatch: 0

Field	Manual/Ground Truth	LLM Extraction	Match
protocol	Smart-seq2	Smart-seq2	✓ Exact
sequencer	Illumina HiSeq 2000	Illumina HiSeq 2000	✓ Exact
tissue	tonsil	tonsil	✓ Exact
cell_type	innate lymphoid cells	Innate lymphoid cells	✓ Exact
organism	human	Homo sapiens	≈ Meaning
experiment_type	single-cell RNA-seq	single-cell RNA-seq	✓ Exact

GSM1811161 (Series: GSE70580)
Evaluable: 6 fields | Exact: 4 | Meaning: 2 | Mismatch: 0

Field	Manual/Ground Truth	LLM Extraction	Match
protocol	Smart-seq2	Smart-seq2	✓ Exact
sequencer	Illumina HiSeq 2000	Illumina HiSeq 2000	✓ Exact
tissue	tonsil	tonsil	✓ Exact
cell_type	innate lymphoid cells	tonsil Innate lymphoid cells	≈ Meaning
organism	human	Homo sapiens	≈ Meaning
experiment_type	single-cell RNA-seq	single-cell RNA-seq	✓ Exact

GSM1811160 (Series: GSE70580)
Evaluable: 6 fields | Exact: 5 | Meaning: 1 | Mismatch: 0

Field	Manual/Ground Truth	LLM Extraction	Match
protocol	Smart-seq2	Smart-seq2	✓ Exact
sequencer	Illumina HiSeq 2000	Illumina HiSeq 2000	✓ Exact
tissue	tonsil	tonsil	✓ Exact
cell_type	innate lymphoid cells	Innate lymphoid cells	✓ Exact
organism	human	Homo sapiens	≈ Meaning
experiment_type	single-cell RNA-seq	single-cell RNA-seq	✓ Exact

GSM1811159 (Series: GSE70580)
Evaluable: 6 fields | Exact: 5 | Meaning: 1 | Mismatch: 0

Field	Manual/Ground Truth	LLM Extraction	Match
protocol	Smart-seq2	Smart-seq2	✓ Exact
sequencer	Illumina HiSeq 2000	Illumina HiSeq 2000	✓ Exact
tissue	tonsil	tonsil	✓ Exact
cell_type	innate lymphoid cells	Innate lymphoid cells	✓ Exact
organism	human	Homo sapiens	≈ Meaning
experiment_type	single-cell RNA-seq	single-cell RNA-seq	✓ Exact

GSM2320928 (Series: GSE87069)
Evaluable: 7 fields | Exact: 2 | Meaning: 4 | Mismatch: 1

Field	Manual/Ground Truth	LLM Extraction	Match
protocol	Smart-seq2	Smart-seq2	✓ Exact
sequencer	Illumina HiSeq 2000	Illumina HiSeq 2000	✓ Exact
tissue	brain	developing ventral midbrain cells	≈ Meaning
organism	mouse	Mus musculus	≈ Meaning
age	E11.5-E13.5	E11.5	≈ Meaning
genotype	wild-type	C57BL/6 Heterozygous Lmx1a-EGFP	✗ Mismatch
experiment_type	single-cell RNA-seq	RNA-Seq	≈ Meaning

GSM2320927 (Series: GSE87069)
Evaluable: 7 fields | Exact: 2 | Meaning: 4 | Mismatch: 1

Field	Manual/Ground Truth	LLM Extraction	Match
protocol	Smart-seq2	Smart-seq2	✓ Exact
sequencer	Illumina HiSeq 2000	Illumina HiSeq 2000	✓ Exact
tissue	brain	developing ventral midbrain cells	≈ Meaning
organism	mouse	Mus musculus	≈ Meaning
age	E11.5-E13.5	E11.5	≈ Meaning
genotype	wild-type	C57BL/6 Heterozygous Lmx1a-EGFP	✗ Mismatch
experiment_type	single-cell RNA-seq	RNA-seq	≈ Meaning

GSM2320926 (Series: GSE87069)
Evaluable: 7 fields | Exact: 2 | Meaning: 4 | Mismatch: 1

Field	Manual/Ground Truth	LLM Extraction	Match
protocol	Smart-seq2	Smart-seq2	✓ Exact
sequencer	Illumina HiSeq 2000	Illumina HiSeq 2000	✓ Exact
tissue	brain	developing ventral midbrain cells	≈ Meaning
organism	mouse	Mus musculus	≈ Meaning
age	E11.5-E13.5	E11.5	≈ Meaning
genotype	wild-type	C57BL/6 Heterozygous Lmx1a-EGFP	✗ Mismatch
experiment_type	single-cell RNA-seq	RNA-Seq	≈ Meaning

GSM2320925 (Series: GSE87069)
Evaluable: 7 fields | Exact: 2 | Meaning: 4 | Mismatch: 1

Field	Manual/Ground Truth	LLM Extraction	Match
protocol	Smart-seq2	Smart-seq2	✓ Exact
sequencer	Illumina HiSeq 2000	Illumina HiSeq 2000	✓ Exact
tissue	brain	developing ventral midbrain cells	≈ Meaning
organism	mouse	Mus musculus	≈ Meaning
age	E11.5-E13.5	E11.5	≈ Meaning
genotype	wild-type	C57BL/6 Heterozygous Lmx1a-EGFP	✗ Mismatch
experiment_type	single-cell RNA-seq	RNA-Seq	≈ Meaning

GSM2320924 (Series: GSE87069)
Evaluable: 7 fields | Exact: 3 | Meaning: 3 | Mismatch: 1

Field	Manual/Ground Truth	LLM Extraction	Match
protocol	Smart-seq2	Smart-seq2	✓ Exact
sequencer	Illumina HiSeq 2000	Illumina HiSeq 2000	✓ Exact
tissue	brain	developing ventral midbrain cells	≈ Meaning
organism	mouse	Mus musculus	≈ Meaning
age	E11.5-E13.5	E11.5	≈ Meaning
genotype	wild-type	C57BL/6 Heterozygous Lmx1a-EGFP	✗ Mismatch
experiment_type	single-cell RNA-seq	single-cell RNA-seq	✓ Exact

GSM2452132 (Series: GSE93374)
Evaluable: 9 fields | Exact: 4 | Meaning: 3 | Mismatch: 2

Field	Manual/Ground Truth	LLM Extraction	Match
protocol	Drop-seq	Drop-seq	✓ Exact
sequencer	Illumina NextSeq 500	Illumina NextSeq 500	✓ Exact
tissue	brain	Arcuate-Median Eminence	≈ Meaning
organism	mouse	Mus musculus	≈ Meaning
age	8-12 weeks	59 days	✗ Mismatch
sex	male	female	≈ Meaning
treatment	fasting	fasting	✓ Exact
genotype	wild-type	C57BL6/J	✗ Mismatch
experiment_type	single-cell RNA-seq	single-cell RNA-seq	✓ Exact

GSM2452131 (Series: GSE93374)
Evaluable: 9 fields | Exact: 4 | Meaning: 3 | Mismatch: 2

Field	Manual/Ground Truth	LLM Extraction	Match
protocol	Drop-seq	Drop-seq	✓ Exact
sequencer	Illumina NextSeq 500	Illumina NextSeq 500	✓ Exact
tissue	brain	Arcuate-Median Eminence	≈ Meaning
organism	mouse	Mus musculus	≈ Meaning
age	8-12 weeks	59 days	✗ Mismatch
sex	male	female	≈ Meaning
treatment	ad libitum feeding	ad libitum feeding	✓ Exact
genotype	wild-type	C57BL6/J	✗ Mismatch
experiment_type	single-cell RNA-seq	single-cell RNA-seq	✓ Exact

GSM2452130 (Series: GSE93374)
Evaluable: 9 fields | Exact: 5 | Meaning: 2 | Mismatch: 2

Field	Manual/Ground Truth	LLM Extraction	Match
protocol	Drop-seq	Drop-seq	✓ Exact
sequencer	Illumina NextSeq 500	Illumina NextSeq 500	✓ Exact
tissue	brain	Arcuate-Median Eminence	≈ Meaning
organism	mouse	Mus musculus	≈ Meaning
age	8-12 weeks	59 days	✗ Mismatch
sex	male	male	✓ Exact
treatment	fasting	fasting	✓ Exact
genotype	wild-type	C57BL6/J	✗ Mismatch
experiment_type	single-cell RNA-seq	single-cell RNA-seq	✓ Exact

GSM2452129 (Series: GSE93374)
Evaluable: 9 fields | Exact: 5 | Meaning: 2 | Mismatch: 2

Field	Manual/Ground Truth	LLM Extraction	Match
protocol	Drop-seq	Drop-seq	✓ Exact
sequencer	Illumina NextSeq 500	Illumina NextSeq 500	✓ Exact
tissue	brain	Arcuate-Median Eminence	≈ Meaning
organism	mouse	Mus musculus	≈ Meaning
age	8-12 weeks	59 days	✗ Mismatch
sex	male	male	✓ Exact
treatment	ad libitum feeding	ad libitum feeding	✓ Exact
genotype	wild-type	C57BL6/J	✗ Mismatch
experiment_type	single-cell RNA-seq	single-cell RNA-seq	✓ Exact

GSM2452128 (Series: GSE93374)
Evaluable: 9 fields | Exact: 3 | Meaning: 2 | Mismatch: 4

Field	Manual/Ground Truth	LLM Extraction	Match
protocol	Drop-seq	Drop-seq	✓ Exact
sequencer	Illumina NextSeq 500	Illumina NextSeq 500	✓ Exact
tissue	brain	Arcuate-Median Eminence	≈ Meaning
organism	mouse	Mus musculus	≈ Meaning
age	8-12 weeks	75 days	✗ Mismatch
sex	male	mixed	✗ Mismatch
treatment	fasting	24 hour fast + 2 hour refeeding	✗ Mismatch
genotype	wild-type	C57BL6/J	✗ Mismatch
experiment_type	single-cell RNA-seq	single-cell RNA-seq	✓ Exact

GSM2913270 (Series: GSE108788)
Evaluable: 8 fields | Exact: 3 | Meaning: 3 | Mismatch: 2

Field	Manual/Ground Truth	LLM Extraction	Match
protocol	Chromium Single Cell 3' v2	Chromium Single Cell 3’ v2	✓ Exact
sequencer	Illumina HiSeq 4000	Illumina HiSeq 4000	✓ Exact
tissue	spinal cord	Cervical and lumbar spinal cord	≈ Meaning
cell_type	V2a interneurons	V2a interneuron	≈ Meaning
organism	mouse	Mus musculus	≈ Meaning
age	P5	P0	✗ Mismatch
genotype	Chx10-Cre	mix of C57BL/6J and CB6F1/J	✗ Mismatch
experiment_type	single-cell RNA-seq	single-cell RNA-seq	✓ Exact

GSM1810510 (Series: GSE70580)
Evaluable: 6 fields | Exact: 6 | Meaning: 0 | Mismatch: 0

Field	Manual/Ground Truth	LLM Extraction	Match
protocol	Smart-seq2	Smart-seq2	✓ Exact
sequencer	Illumina HiSeq 2000	Illumina HiSeq 2000	✓ Exact
tissue	tonsil	tonsil	✓ Exact
cell_type	Innate lymphoid cells	Innate lymphoid cells	✓ Exact
organism	Homo sapiens	Homo sapiens	✓ Exact
experiment_type	single-cell RNA-seq	single-cell RNA-seq	✓ Exact

GSM1810526 (Series: GSE70580)
Evaluable: 6 fields | Exact: 6 | Meaning: 0 | Mismatch: 0

Field	Manual/Ground Truth	LLM Extraction	Match
protocol	Smart-seq2	Smart-seq2	✓ Exact
sequencer	Illumina HiSeq 2000	Illumina HiSeq 2000	✓ Exact
tissue	tonsil	tonsil	✓ Exact
cell_type	Innate lymphoid cells	Innate lymphoid cells	✓ Exact
organism	Homo sapiens	Homo sapiens	✓ Exact
experiment_type	single-cell RNA-seq	single-cell RNA-seq	✓ Exact

GSM1810542 (Series: GSE70580)
Evaluable: 6 fields | Exact: 6 | Meaning: 0 | Mismatch: 0

Field	Manual/Ground Truth	LLM Extraction	Match
protocol	Smart-seq2	Smart-seq2	✓ Exact
sequencer	Illumina HiSeq 2000	Illumina HiSeq 2000	✓ Exact
tissue	tonsil	tonsil	✓ Exact
cell_type	tonsil Innate lymphoid cells	tonsil Innate lymphoid cells	✓ Exact
organism	Homo sapiens	Homo sapiens	✓ Exact
experiment_type	single-cell RNA-seq	single-cell RNA-seq	✓ Exact

GSM1810558 (Series: GSE70580)
Evaluable: 6 fields | Exact: 6 | Meaning: 0 | Mismatch: 0

Field	Manual/Ground Truth	LLM Extraction	Match
protocol	Smart-seq2	Smart-seq2	✓ Exact
sequencer	Illumina HiSeq 2000	Illumina HiSeq 2000	✓ Exact
tissue	tonsil	tonsil	✓ Exact
cell_type	Innate lymphoid cells	Innate lymphoid cells	✓ Exact
organism	Homo sapiens	Homo sapiens	✓ Exact
experiment_type	single-cell RNA-seq	single-cell RNA-seq	✓ Exact

GSM1810574 (Series: GSE70580)
Evaluable: 6 fields | Exact: 6 | Meaning: 0 | Mismatch: 0

Field	Manual/Ground Truth	LLM Extraction	Match
protocol	Smart-seq2	Smart-seq2	✓ Exact
sequencer	Illumina HiSeq 2000	Illumina HiSeq 2000	✓ Exact
tissue	tonsil	tonsil	✓ Exact
cell_type	tonsil Innate lymphoid cells	tonsil Innate lymphoid cells	✓ Exact
organism	Homo sapiens	Homo sapiens	✓ Exact
experiment_type	single-cell RNA-seq	single-cell RNA-seq	✓ Exact

GSM1810590 (Series: GSE70580)
Evaluable: 6 fields | Exact: 6 | Meaning: 0 | Mismatch: 0

Field	Manual/Ground Truth	LLM Extraction	Match
protocol	Smart-seq2	Smart-seq2	✓ Exact
sequencer	Illumina HiSeq 2000	Illumina HiSeq 2000	✓ Exact
tissue	tonsil	tonsil	✓ Exact
cell_type	tonsil Innate lymphoid cells	tonsil Innate lymphoid cells	✓ Exact
organism	Homo sapiens	Homo sapiens	✓ Exact
experiment_type	single-cell RNA-seq	single-cell RNA-seq	✓ Exact

GSM1810606 (Series: GSE70580)
Evaluable: 6 fields | Exact: 6 | Meaning: 0 | Mismatch: 0

Field	Manual/Ground Truth	LLM Extraction	Match
protocol	Smart-seq2	Smart-seq2	✓ Exact
sequencer	Illumina HiSeq 2000	Illumina HiSeq 2000	✓ Exact
tissue	tonsil	tonsil	✓ Exact
cell_type	Innate lymphoid cells	Innate lymphoid cells	✓ Exact
organism	Homo sapiens	Homo sapiens	✓ Exact
experiment_type	single-cell RNA-seq	single-cell RNA-seq	✓ Exact

GSM1810622 (Series: GSE70580)
Evaluable: 6 fields | Exact: 6 | Meaning: 0 | Mismatch: 0

Field	Manual/Ground Truth	LLM Extraction	Match
protocol	Smart-seq2	Smart-seq2	✓ Exact
sequencer	Illumina HiSeq 2000	Illumina HiSeq 2000	✓ Exact
tissue	tonsil	tonsil	✓ Exact
cell_type	Innate lymphoid cells	Innate lymphoid cells	✓ Exact
organism	Homo sapiens	Homo sapiens	✓ Exact
experiment_type	single-cell RNA-seq	single-cell RNA-seq	✓ Exact

GSM1810638 (Series: GSE70580)
Evaluable: 6 fields | Exact: 6 | Meaning: 0 | Mismatch: 0

Field	Manual/Ground Truth	LLM Extraction	Match
protocol	Smart-seq2	Smart-seq2	✓ Exact
sequencer	Illumina HiSeq 2000	Illumina HiSeq 2000	✓ Exact
tissue	tonsil	tonsil	✓ Exact
cell_type	tonsil Innate lymphoid cells	tonsil Innate lymphoid cells	✓ Exact
organism	Homo sapiens	Homo sapiens	✓ Exact
experiment_type	single-cell RNA-seq	single-cell RNA-seq	✓ Exact

GSM1810654 (Series: GSE70580)
Evaluable: 6 fields | Exact: 6 | Meaning: 0 | Mismatch: 0

Field	Manual/Ground Truth	LLM Extraction	Match
protocol	Smart-seq2	Smart-seq2	✓ Exact
sequencer	Illumina HiSeq 2000	Illumina HiSeq 2000	✓ Exact
tissue	tonsil	tonsil	✓ Exact
cell_type	Innate lymphoid cells	Innate lymphoid cells	✓ Exact
organism	Homo sapiens	Homo sapiens	✓ Exact
experiment_type	single-cell RNA-seq	single-cell RNA-seq	✓ Exact

GSM1810670 (Series: GSE70580)
Evaluable: 6 fields | Exact: 6 | Meaning: 0 | Mismatch: 0

Field	Manual/Ground Truth	LLM Extraction	Match
protocol	Smart-seq2	Smart-seq2	✓ Exact
sequencer	Illumina HiSeq 2000	Illumina HiSeq 2000	✓ Exact
tissue	tonsil	tonsil	✓ Exact
cell_type	Innate lymphoid cells	Innate lymphoid cells	✓ Exact
organism	Homo sapiens	Homo sapiens	✓ Exact
experiment_type	single-cell RNA-seq	single-cell RNA-seq	✓ Exact

GSM1810686 (Series: GSE70580)
Evaluable: 6 fields | Exact: 6 | Meaning: 0 | Mismatch: 0

Field	Manual/Ground Truth	LLM Extraction	Match
protocol	Smart-seq2	Smart-seq2	✓ Exact
sequencer	Illumina HiSeq 2000	Illumina HiSeq 2000	✓ Exact
tissue	tonsil	tonsil	✓ Exact
cell_type	Innate lymphoid cells	Innate lymphoid cells	✓ Exact
organism	Homo sapiens	Homo sapiens	✓ Exact
experiment_type	single-cell RNA-seq	single-cell RNA-seq	✓ Exact

GSM1810702 (Series: GSE70580)
Evaluable: 6 fields | Exact: 6 | Meaning: 0 | Mismatch: 0

Field	Manual/Ground Truth	LLM Extraction	Match
protocol	Smart-seq2	Smart-seq2	✓ Exact
sequencer	Illumina HiSeq 2000	Illumina HiSeq 2000	✓ Exact
tissue	tonsil	tonsil	✓ Exact
cell_type	tonsil Innate lymphoid cells	tonsil Innate lymphoid cells	✓ Exact
organism	Homo sapiens	Homo sapiens	✓ Exact
experiment_type	single-cell RNA-seq	single-cell RNA-seq	✓ Exact

GSM1810718 (Series: GSE70580)
Evaluable: 6 fields | Exact: 6 | Meaning: 0 | Mismatch: 0

Field	Manual/Ground Truth	LLM Extraction	Match
protocol	Smart-seq2	Smart-seq2	✓ Exact
sequencer	Illumina HiSeq 2000	Illumina HiSeq 2000	✓ Exact
tissue	tonsil	tonsil	✓ Exact
cell_type	tonsil Innate lymphoid cells	tonsil Innate lymphoid cells	✓ Exact
organism	Homo sapiens	Homo sapiens	✓ Exact
experiment_type	single-cell RNA-seq	single-cell RNA-seq	✓ Exact

GSM1810734 (Series: GSE70580)
Evaluable: 6 fields | Exact: 6 | Meaning: 0 | Mismatch: 0

Field	Manual/Ground Truth	LLM Extraction	Match
protocol	Smart-seq2	Smart-seq2	✓ Exact
sequencer	Illumina HiSeq 2000	Illumina HiSeq 2000	✓ Exact
tissue	tonsil	tonsil	✓ Exact
cell_type	tonsil Innate lymphoid cells	tonsil Innate lymphoid cells	✓ Exact
organism	Homo sapiens	Homo sapiens	✓ Exact
experiment_type	single-cell RNA-seq	single-cell RNA-seq	✓ Exact

GSM1810750 (Series: GSE70580)
Evaluable: 6 fields | Exact: 6 | Meaning: 0 | Mismatch: 0

Field	Manual/Ground Truth	LLM Extraction	Match
protocol	Smart-seq2	Smart-seq2	✓ Exact
sequencer	Illumina HiSeq 2000	Illumina HiSeq 2000	✓ Exact
tissue	tonsil	tonsil	✓ Exact
cell_type	Innate lymphoid cells	Innate lymphoid cells	✓ Exact
organism	Homo sapiens	Homo sapiens	✓ Exact
experiment_type	single-cell RNA-seq	single-cell RNA-seq	✓ Exact

GSM1810766 (Series: GSE70580)
Evaluable: 6 fields | Exact: 6 | Meaning: 0 | Mismatch: 0

Field	Manual/Ground Truth	LLM Extraction	Match
protocol	Smart-seq2	Smart-seq2	✓ Exact
sequencer	Illumina HiSeq 2000	Illumina HiSeq 2000	✓ Exact
tissue	tonsil	tonsil	✓ Exact
cell_type	Innate lymphoid cells	Innate lymphoid cells	✓ Exact
organism	Homo sapiens	Homo sapiens	✓ Exact
experiment_type	single-cell RNA-seq	single-cell RNA-seq	✓ Exact

GSM1810782 (Series: GSE70580)
Evaluable: 6 fields | Exact: 6 | Meaning: 0 | Mismatch: 0

Field	Manual/Ground Truth	LLM Extraction	Match
protocol	Smart-seq2	Smart-seq2	✓ Exact
sequencer	Illumina HiSeq 2000	Illumina HiSeq 2000	✓ Exact
tissue	tonsil	tonsil	✓ Exact
cell_type	tonsil Innate lymphoid cells	tonsil Innate lymphoid cells	✓ Exact
organism	Homo sapiens	Homo sapiens	✓ Exact
experiment_type	single-cell RNA-seq	single-cell RNA-seq	✓ Exact

GSM1810798 (Series: GSE70580)
Evaluable: 6 fields | Exact: 6 | Meaning: 0 | Mismatch: 0

Field	Manual/Ground Truth	LLM Extraction	Match
protocol	Smart-seq2	Smart-seq2	✓ Exact
sequencer	Illumina HiSeq 2000	Illumina HiSeq 2000	✓ Exact
tissue	tonsil	tonsil	✓ Exact
cell_type	Innate lymphoid cells	Innate lymphoid cells	✓ Exact
organism	Homo sapiens	Homo sapiens	✓ Exact
experiment_type	single-cell RNA-seq	single-cell RNA-seq	✓ Exact

GSM1810814 (Series: GSE70580)
Evaluable: 6 fields | Exact: 6 | Meaning: 0 | Mismatch: 0

Field	Manual/Ground Truth	LLM Extraction	Match
protocol	Smart-seq2	Smart-seq2	✓ Exact
sequencer	Illumina HiSeq 2000	Illumina HiSeq 2000	✓ Exact
tissue	tonsil	tonsil	✓ Exact
cell_type	Innate lymphoid cells	Innate lymphoid cells	✓ Exact
organism	Homo sapiens	Homo sapiens	✓ Exact
experiment_type	single-cell RNA-seq	single-cell RNA-seq	✓ Exact

GSM1810830 (Series: GSE70580)
Evaluable: 6 fields | Exact: 6 | Meaning: 0 | Mismatch: 0

Field	Manual/Ground Truth	LLM Extraction	Match
protocol	Smart-seq2	Smart-seq2	✓ Exact
sequencer	Illumina HiSeq 2000	Illumina HiSeq 2000	✓ Exact
tissue	tonsil	tonsil	✓ Exact
cell_type	Innate lymphoid cells	Innate lymphoid cells	✓ Exact
organism	Homo sapiens	Homo sapiens	✓ Exact
experiment_type	single-cell RNA-seq	single-cell RNA-seq	✓ Exact

GSM1810846 (Series: GSE70580)
Evaluable: 6 fields | Exact: 6 | Meaning: 0 | Mismatch: 0

Field	Manual/Ground Truth	LLM Extraction	Match
protocol	Smart-seq2	Smart-seq2	✓ Exact
sequencer	Illumina HiSeq 2000	Illumina HiSeq 2000	✓ Exact
tissue	tonsil	tonsil	✓ Exact
cell_type	Innate lymphoid cells	Innate lymphoid cells	✓ Exact
organism	Homo sapiens	Homo sapiens	✓ Exact
experiment_type	single-cell RNA-seq	single-cell RNA-seq	✓ Exact

GSM1810862 (Series: GSE70580)
Evaluable: 6 fields | Exact: 6 | Meaning: 0 | Mismatch: 0

Field	Manual/Ground Truth	LLM Extraction	Match
protocol	Smart-seq2	Smart-seq2	✓ Exact
sequencer	Illumina HiSeq 2000	Illumina HiSeq 2000	✓ Exact
tissue	tonsil	tonsil	✓ Exact
cell_type	tonsil Innate lymphoid cells	tonsil Innate lymphoid cells	✓ Exact
organism	Homo sapiens	Homo sapiens	✓ Exact
experiment_type	single-cell RNA-seq	single-cell RNA-seq	✓ Exact

GSM1810878 (Series: GSE70580)
Evaluable: 6 fields | Exact: 6 | Meaning: 0 | Mismatch: 0

Field	Manual/Ground Truth	LLM Extraction	Match
protocol	Smart-seq2	Smart-seq2	✓ Exact
sequencer	Illumina HiSeq 2000	Illumina HiSeq 2000	✓ Exact
tissue	tonsil	tonsil	✓ Exact
cell_type	Innate lymphoid cells	Innate lymphoid cells	✓ Exact
organism	Homo sapiens	Homo sapiens	✓ Exact
experiment_type	single-cell RNA-seq	single-cell RNA-seq	✓ Exact

GSM1810894 (Series: GSE70580)
Evaluable: 6 fields | Exact: 6 | Meaning: 0 | Mismatch: 0

Field	Manual/Ground Truth	LLM Extraction	Match
protocol	Smart-seq2	Smart-seq2	✓ Exact
sequencer	Illumina HiSeq 2000	Illumina HiSeq 2000	✓ Exact
tissue	tonsil	tonsil	✓ Exact
cell_type	tonsil Innate lymphoid cells	tonsil Innate lymphoid cells	✓ Exact
organism	Homo sapiens	Homo sapiens	✓ Exact
experiment_type	single-cell RNA-seq	single-cell RNA-seq	✓ Exact

GSM1810910 (Series: GSE70580)
Evaluable: 6 fields | Exact: 6 | Meaning: 0 | Mismatch: 0

Field	Manual/Ground Truth	LLM Extraction	Match
protocol	Smart-seq2	Smart-seq2	✓ Exact
sequencer	Illumina HiSeq 2000	Illumina HiSeq 2000	✓ Exact
tissue	tonsil	tonsil	✓ Exact
cell_type	tonsil Innate lymphoid cells	tonsil Innate lymphoid cells	✓ Exact
organism	Homo sapiens	Homo sapiens	✓ Exact
experiment_type	single-cell RNA-seq	single-cell RNA-seq	✓ Exact

GSM1810926 (Series: GSE70580)
Evaluable: 6 fields | Exact: 6 | Meaning: 0 | Mismatch: 0

Field	Manual/Ground Truth	LLM Extraction	Match
protocol	Smart-seq2	Smart-seq2	✓ Exact
sequencer	Illumina HiSeq 2000	Illumina HiSeq 2000	✓ Exact
tissue	tonsil	tonsil	✓ Exact
cell_type	tonsil Innate lymphoid cells	tonsil Innate lymphoid cells	✓ Exact
organism	Homo sapiens	Homo sapiens	✓ Exact
experiment_type	single-cell RNA-seq	single-cell RNA-seq	✓ Exact

GSM1810942 (Series: GSE70580)
Evaluable: 6 fields | Exact: 6 | Meaning: 0 | Mismatch: 0

Field	Manual/Ground Truth	LLM Extraction	Match
protocol	Smart-seq2	Smart-seq2	✓ Exact
sequencer	Illumina HiSeq 2000	Illumina HiSeq 2000	✓ Exact
tissue	tonsil	tonsil	✓ Exact
cell_type	Innate lymphoid cells	Innate lymphoid cells	✓ Exact
organism	Homo sapiens	Homo sapiens	✓ Exact
experiment_type	single-cell RNA-seq	single-cell RNA-seq	✓ Exact

GSM1810958 (Series: GSE70580)
Evaluable: 6 fields | Exact: 6 | Meaning: 0 | Mismatch: 0

Field	Manual/Ground Truth	LLM Extraction	Match
protocol	Smart-seq2	Smart-seq2	✓ Exact
sequencer	Illumina HiSeq 2000	Illumina HiSeq 2000	✓ Exact
tissue	tonsil	tonsil	✓ Exact
cell_type	tonsil Innate lymphoid cells	tonsil Innate lymphoid cells	✓ Exact
organism	Homo sapiens	Homo sapiens	✓ Exact
experiment_type	single-cell RNA-seq	single-cell RNA-seq	✓ Exact

GSM1810974 (Series: GSE70580)
Evaluable: 6 fields | Exact: 6 | Meaning: 0 | Mismatch: 0

Field	Manual/Ground Truth	LLM Extraction	Match
protocol	Smart-seq2	Smart-seq2	✓ Exact
sequencer	Illumina HiSeq 2000	Illumina HiSeq 2000	✓ Exact
tissue	tonsil	tonsil	✓ Exact
cell_type	Innate lymphoid cells	Innate lymphoid cells	✓ Exact
organism	Homo sapiens	Homo sapiens	✓ Exact
experiment_type	single-cell RNA-seq	single-cell RNA-seq	✓ Exact

GSM1810996 (Series: GSE70580)
Evaluable: 6 fields | Exact: 6 | Meaning: 0 | Mismatch: 0

Field	Manual/Ground Truth	LLM Extraction	Match
protocol	Smart-seq2	Smart-seq2	✓ Exact
sequencer	Illumina HiSeq 2000	Illumina HiSeq 2000	✓ Exact
tissue	tonsil	tonsil	✓ Exact
cell_type	Innate lymphoid cells	Innate lymphoid cells	✓ Exact
organism	Homo sapiens	Homo sapiens	✓ Exact
experiment_type	single-cell RNA-seq	single-cell RNA-seq	✓ Exact

GSM1811012 (Series: GSE70580)
Evaluable: 6 fields | Exact: 6 | Meaning: 0 | Mismatch: 0

Field	Manual/Ground Truth	LLM Extraction	Match
protocol	Smart-seq2	Smart-seq2	✓ Exact
sequencer	Illumina HiSeq 2000	Illumina HiSeq 2000	✓ Exact
tissue	tonsil	tonsil	✓ Exact
cell_type	Innate lymphoid cells	Innate lymphoid cells	✓ Exact
organism	Homo sapiens	Homo sapiens	✓ Exact
experiment_type	single-cell RNA-seq	single-cell RNA-seq	✓ Exact

GSM1811028 (Series: GSE70580)
Evaluable: 6 fields | Exact: 6 | Meaning: 0 | Mismatch: 0

Field	Manual/Ground Truth	LLM Extraction	Match
protocol	Smart-seq2	Smart-seq2	✓ Exact
sequencer	Illumina HiSeq 2000	Illumina HiSeq 2000	✓ Exact
tissue	tonsil	tonsil	✓ Exact
cell_type	Innate lymphoid cells	Innate lymphoid cells	✓ Exact
organism	Homo sapiens	Homo sapiens	✓ Exact
experiment_type	single-cell RNA-seq	single-cell RNA-seq	✓ Exact

GSM1811044 (Series: GSE70580)
Evaluable: 6 fields | Exact: 6 | Meaning: 0 | Mismatch: 0

Field	Manual/Ground Truth	LLM Extraction	Match
protocol	Smart-seq2	Smart-seq2	✓ Exact
sequencer	Illumina HiSeq 2000	Illumina HiSeq 2000	✓ Exact
tissue	tonsil	tonsil	✓ Exact
cell_type	Innate lymphoid cells	Innate lymphoid cells	✓ Exact
organism	Homo sapiens	Homo sapiens	✓ Exact
experiment_type	single-cell RNA-seq	single-cell RNA-seq	✓ Exact

GSM1811060 (Series: GSE70580)
Evaluable: 6 fields | Exact: 6 | Meaning: 0 | Mismatch: 0

Field	Manual/Ground Truth	LLM Extraction	Match
protocol	Smart-seq2	Smart-seq2	✓ Exact
sequencer	Illumina HiSeq 2000	Illumina HiSeq 2000	✓ Exact
tissue	tonsil	tonsil	✓ Exact
cell_type	tonsil Innate lymphoid cells	tonsil Innate lymphoid cells	✓ Exact
organism	Homo sapiens	Homo sapiens	✓ Exact
experiment_type	single-cell RNA-seq	single-cell RNA-seq	✓ Exact

GSM1811076 (Series: GSE70580)
Evaluable: 6 fields | Exact: 6 | Meaning: 0 | Mismatch: 0

Field	Manual/Ground Truth	LLM Extraction	Match
protocol	Smart-seq2	Smart-seq2	✓ Exact
sequencer	Illumina HiSeq 2000	Illumina HiSeq 2000	✓ Exact
tissue	tonsil	tonsil	✓ Exact
cell_type	tonsil Innate lymphoid cells	tonsil Innate lymphoid cells	✓ Exact
organism	Homo sapiens	Homo sapiens	✓ Exact
experiment_type	single-cell RNA-seq	single-cell RNA-seq	✓ Exact

GSM1811092 (Series: GSE70580)
Evaluable: 6 fields | Exact: 6 | Meaning: 0 | Mismatch: 0

Field	Manual/Ground Truth	LLM Extraction	Match
protocol	Smart-seq2	Smart-seq2	✓ Exact
sequencer	Illumina HiSeq 2000	Illumina HiSeq 2000	✓ Exact
tissue	tonsil	tonsil	✓ Exact
cell_type	tonsil Innate lymphoid cells	tonsil Innate lymphoid cells	✓ Exact
organism	Homo sapiens	Homo sapiens	✓ Exact
experiment_type	single-cell RNA-seq	single-cell RNA-seq	✓ Exact

GSM1811108 (Series: GSE70580)
Evaluable: 6 fields | Exact: 6 | Meaning: 0 | Mismatch: 0

Field	Manual/Ground Truth	LLM Extraction	Match
protocol	Smart-seq2	Smart-seq2	✓ Exact
sequencer	Illumina HiSeq 2000	Illumina HiSeq 2000	✓ Exact
tissue	tonsil	tonsil	✓ Exact
cell_type	tonsil Innate lymphoid cells	tonsil Innate lymphoid cells	✓ Exact
organism	Homo sapiens	Homo sapiens	✓ Exact
experiment_type	single-cell RNA-seq	single-cell RNA-seq	✓ Exact

GSM1811124 (Series: GSE70580)
Evaluable: 6 fields | Exact: 6 | Meaning: 0 | Mismatch: 0

Field	Manual/Ground Truth	LLM Extraction	Match
protocol	Smart-seq2	Smart-seq2	✓ Exact
sequencer	Illumina HiSeq 2000	Illumina HiSeq 2000	✓ Exact
tissue	tonsil	tonsil	✓ Exact
cell_type	Innate lymphoid cells	Innate lymphoid cells	✓ Exact
organism	Homo sapiens	Homo sapiens	✓ Exact
experiment_type	single-cell RNA-seq	single-cell RNA-seq	✓ Exact

GSM1811140 (Series: GSE70580)
Evaluable: 6 fields | Exact: 6 | Meaning: 0 | Mismatch: 0

Field	Manual/Ground Truth	LLM Extraction	Match
protocol	Smart-seq2	Smart-seq2	✓ Exact
sequencer	Illumina HiSeq 2000	Illumina HiSeq 2000	✓ Exact
tissue	tonsil	tonsil	✓ Exact
cell_type	tonsil Innate lymphoid cells	tonsil Innate lymphoid cells	✓ Exact
organism	Homo sapiens	Homo sapiens	✓ Exact
experiment_type	single-cell RNA-seq	single-cell RNA-seq	✓ Exact

GSM2320355 (Series: GSE87069)
Evaluable: 7 fields | Exact: 7 | Meaning: 0 | Mismatch: 0

Field	Manual/Ground Truth	LLM Extraction	Match
protocol	Smart-seq2	Smart-seq2	✓ Exact
sequencer	Illumina HiSeq 2000	Illumina HiSeq 2000	✓ Exact
tissue	developing ventral midbrain cells	developing ventral midbrain cells	✓ Exact
organism	Mus musculus	Mus musculus	✓ Exact
age	E12.5	E12.5	✓ Exact
genotype	C57BL/6 Heterozygous Lmx1a-EGFP	C57BL/6 Heterozygous Lmx1a-EGFP	✓ Exact
experiment_type	single-cell RNA-seq	single-cell RNA-seq	✓ Exact

GSM2320369 (Series: GSE87069)
Evaluable: 7 fields | Exact: 7 | Meaning: 0 | Mismatch: 0

Field	Manual/Ground Truth	LLM Extraction	Match
protocol	Smart-seq2	Smart-seq2	✓ Exact
sequencer	Illumina HiSeq 2000	Illumina HiSeq 2000	✓ Exact
tissue	developing ventral midbrain cells	developing ventral midbrain cells	✓ Exact
organism	Mus musculus	Mus musculus	✓ Exact
age	E12.5	E12.5	✓ Exact
genotype	C57BL/6 Heterozygous Lmx1a-EGFP	C57BL/6 Heterozygous Lmx1a-EGFP	✓ Exact
experiment_type	single-cell RNA-seq	single-cell RNA-seq	✓ Exact

GSM2320383 (Series: GSE87069)
Evaluable: 7 fields | Exact: 7 | Meaning: 0 | Mismatch: 0

Field	Manual/Ground Truth	LLM Extraction	Match
protocol	Smart-seq2	Smart-seq2	✓ Exact
sequencer	Illumina HiSeq 2000	Illumina HiSeq 2000	✓ Exact
tissue	developing ventral midbrain cells	developing ventral midbrain cells	✓ Exact
organism	Mus musculus	Mus musculus	✓ Exact
age	E12.5	E12.5	✓ Exact
genotype	C57BL/6 Heterozygous Lmx1a-EGFP	C57BL/6 Heterozygous Lmx1a-EGFP	✓ Exact
experiment_type	single-cell RNA-seq	single-cell RNA-seq	✓ Exact

GSM2320397 (Series: GSE87069)
Evaluable: 7 fields | Exact: 7 | Meaning: 0 | Mismatch: 0

Field	Manual/Ground Truth	LLM Extraction	Match
protocol	Smart-seq2	Smart-seq2	✓ Exact
sequencer	Illumina HiSeq 2000	Illumina HiSeq 2000	✓ Exact
tissue	developing ventral midbrain cells	developing ventral midbrain cells	✓ Exact
organism	Mus musculus	Mus musculus	✓ Exact
age	E12.5	E12.5	✓ Exact
genotype	C57BL/6 Heterozygous Lmx1a-EGFP	C57BL/6 Heterozygous Lmx1a-EGFP	✓ Exact
experiment_type	single-cell RNA-seq	single-cell RNA-seq	✓ Exact

GSM2320411 (Series: GSE87069)
Evaluable: 7 fields | Exact: 7 | Meaning: 0 | Mismatch: 0

Field	Manual/Ground Truth	LLM Extraction	Match
protocol	Smart-seq2	Smart-seq2	✓ Exact
sequencer	Illumina HiSeq 2000	Illumina HiSeq 2000	✓ Exact
tissue	developing ventral midbrain cells	developing ventral midbrain cells	✓ Exact
organism	Mus musculus	Mus musculus	✓ Exact
age	E12.5	E12.5	✓ Exact
genotype	C57BL/6 Heterozygous Lmx1a-EGFP	C57BL/6 Heterozygous Lmx1a-EGFP	✓ Exact
experiment_type	single-cell RNA-seq	single-cell RNA-seq	✓ Exact

GSM2320425 (Series: GSE87069)
Evaluable: 7 fields | Exact: 7 | Meaning: 0 | Mismatch: 0

Field	Manual/Ground Truth	LLM Extraction	Match
protocol	Smart-seq2	Smart-seq2	✓ Exact
sequencer	Illumina HiSeq 2000	Illumina HiSeq 2000	✓ Exact
tissue	developing ventral midbrain cells	developing ventral midbrain cells	✓ Exact
organism	Mus musculus	Mus musculus	✓ Exact
age	E10.5	E10.5	✓ Exact
genotype	C57BL/6 Heterozygous Lmx1a-EGFP	C57BL/6 Heterozygous Lmx1a-EGFP	✓ Exact
experiment_type	single-cell RNA-seq	single-cell RNA-seq	✓ Exact

GSM2320439 (Series: GSE87069)
Evaluable: 7 fields | Exact: 7 | Meaning: 0 | Mismatch: 0

Field	Manual/Ground Truth	LLM Extraction	Match
protocol	Smart-seq2	Smart-seq2	✓ Exact
sequencer	Illumina HiSeq 2000	Illumina HiSeq 2000	✓ Exact
tissue	developing ventral midbrain cells	developing ventral midbrain cells	✓ Exact
organism	Mus musculus	Mus musculus	✓ Exact
age	E10.5	E10.5	✓ Exact
genotype	C57BL/6 Heterozygous Lmx1a-EGFP	C57BL/6 Heterozygous Lmx1a-EGFP	✓ Exact
experiment_type	RNA-seq	RNA-seq	✓ Exact

GSM2320453 (Series: GSE87069)
Evaluable: 7 fields | Exact: 7 | Meaning: 0 | Mismatch: 0

Field	Manual/Ground Truth	LLM Extraction	Match
protocol	Smart-seq2	Smart-seq2	✓ Exact
sequencer	Illumina HiSeq 2000	Illumina HiSeq 2000	✓ Exact
tissue	developing ventral midbrain cells	developing ventral midbrain cells	✓ Exact
organism	Mus musculus	Mus musculus	✓ Exact
age	E10.5	E10.5	✓ Exact
genotype	C57BL/6 Heterozygous Lmx1a-EGFP	C57BL/6 Heterozygous Lmx1a-EGFP	✓ Exact
experiment_type	single-cell RNA-seq	single-cell RNA-seq	✓ Exact

GSM2320467 (Series: GSE87069)
Evaluable: 7 fields | Exact: 7 | Meaning: 0 | Mismatch: 0

Field	Manual/Ground Truth	LLM Extraction	Match
protocol	Smart-seq2	Smart-seq2	✓ Exact
sequencer	Illumina HiSeq 2000	Illumina HiSeq 2000	✓ Exact
tissue	developing ventral midbrain cells	developing ventral midbrain cells	✓ Exact
organism	Mus musculus	Mus musculus	✓ Exact
age	E10.5	E10.5	✓ Exact
genotype	C57BL/6 Heterozygous Lmx1a-EGFP	C57BL/6 Heterozygous Lmx1a-EGFP	✓ Exact
experiment_type	single-cell RNA-seq	single-cell RNA-seq	✓ Exact

GSM2320481 (Series: GSE87069)
Evaluable: 7 fields | Exact: 7 | Meaning: 0 | Mismatch: 0

Field	Manual/Ground Truth	LLM Extraction	Match
protocol	Smart-seq2	Smart-seq2	✓ Exact
sequencer	Illumina HiSeq 2000	Illumina HiSeq 2000	✓ Exact
tissue	developing ventral midbrain cells	developing ventral midbrain cells	✓ Exact
organism	Mus musculus	Mus musculus	✓ Exact
age	E10.5	E10.5	✓ Exact
genotype	C57BL/6 Heterozygous Lmx1a-EGFP	C57BL/6 Heterozygous Lmx1a-EGFP	✓ Exact
experiment_type	RNA-seq	RNA-seq	✓ Exact

GSM2320495 (Series: GSE87069)
Evaluable: 7 fields | Exact: 7 | Meaning: 0 | Mismatch: 0

Field	Manual/Ground Truth	LLM Extraction	Match
protocol	Smart-seq2	Smart-seq2	✓ Exact
sequencer	Illumina HiSeq 2000	Illumina HiSeq 2000	✓ Exact
tissue	developing ventral midbrain cells	developing ventral midbrain cells	✓ Exact
organism	Mus musculus	Mus musculus	✓ Exact
age	E10.5	E10.5	✓ Exact
genotype	C57BL/6 Heterozygous Lmx1a-EGFP	C57BL/6 Heterozygous Lmx1a-EGFP	✓ Exact
experiment_type	RNA-seq	RNA-seq	✓ Exact

GSM2320509 (Series: GSE87069)
Evaluable: 7 fields | Exact: 7 | Meaning: 0 | Mismatch: 0

Field	Manual/Ground Truth	LLM Extraction	Match
protocol	Smart-seq2	Smart-seq2	✓ Exact
sequencer	Illumina HiSeq 2000	Illumina HiSeq 2000	✓ Exact
tissue	developing ventral midbrain cells	developing ventral midbrain cells	✓ Exact
organism	Mus musculus	Mus musculus	✓ Exact
age	E10.5	E10.5	✓ Exact
genotype	C57BL/6 Heterozygous Lmx1a-EGFP	C57BL/6 Heterozygous Lmx1a-EGFP	✓ Exact
experiment_type	single-cell RNA-seq	single-cell RNA-seq	✓ Exact

GSM2320523 (Series: GSE87069)
Evaluable: 7 fields | Exact: 7 | Meaning: 0 | Mismatch: 0

Field	Manual/Ground Truth	LLM Extraction	Match
protocol	Smart-seq2	Smart-seq2	✓ Exact
sequencer	Illumina HiSeq 2000	Illumina HiSeq 2000	✓ Exact
tissue	developing ventral midbrain cells	developing ventral midbrain cells	✓ Exact
organism	Mus musculus	Mus musculus	✓ Exact
age	E10.5	E10.5	✓ Exact
genotype	C57BL/6 Heterozygous Lmx1a-EGFP	C57BL/6 Heterozygous Lmx1a-EGFP	✓ Exact
experiment_type	single-cell RNA-seq	single-cell RNA-seq	✓ Exact

GSM2320537 (Series: GSE87069)
Evaluable: 7 fields | Exact: 7 | Meaning: 0 | Mismatch: 0

Field	Manual/Ground Truth	LLM Extraction	Match
protocol	Smart-seq2	Smart-seq2	✓ Exact
sequencer	Illumina HiSeq 2000	Illumina HiSeq 2000	✓ Exact
tissue	developing ventral midbrain cells	developing ventral midbrain cells	✓ Exact
organism	Mus musculus	Mus musculus	✓ Exact
age	E10.5	E10.5	✓ Exact
genotype	C57BL/6 Heterozygous Lmx1a-EGFP	C57BL/6 Heterozygous Lmx1a-EGFP	✓ Exact
experiment_type	RNA-Seq	RNA-Seq	✓ Exact

GSM2320551 (Series: GSE87069)
Evaluable: 7 fields | Exact: 7 | Meaning: 0 | Mismatch: 0

Field	Manual/Ground Truth	LLM Extraction	Match
protocol	Smart-seq2	Smart-seq2	✓ Exact
sequencer	Illumina HiSeq 2000	Illumina HiSeq 2000	✓ Exact
tissue	developing ventral midbrain cells	developing ventral midbrain cells	✓ Exact
organism	Mus musculus	Mus musculus	✓ Exact
age	E10.5	E10.5	✓ Exact
genotype	C57BL/6 Heterozygous Lmx1a-EGFP	C57BL/6 Heterozygous Lmx1a-EGFP	✓ Exact
experiment_type	single-cell RNA-seq	single-cell RNA-seq	✓ Exact

GSM2320565 (Series: GSE87069)
Evaluable: 7 fields | Exact: 7 | Meaning: 0 | Mismatch: 0

Field	Manual/Ground Truth	LLM Extraction	Match
protocol	Smart-seq2	Smart-seq2	✓ Exact
sequencer	Illumina HiSeq 2000	Illumina HiSeq 2000	✓ Exact
tissue	developing ventral midbrain cells	developing ventral midbrain cells	✓ Exact
organism	Mus musculus	Mus musculus	✓ Exact
age	E12.5	E12.5	✓ Exact
genotype	C57BL/6 Heterozygous Lmx1a-EGFP	C57BL/6 Heterozygous Lmx1a-EGFP	✓ Exact
experiment_type	single-cell RNA-seq	single-cell RNA-seq	✓ Exact

GSM2320579 (Series: GSE87069)
Evaluable: 7 fields | Exact: 7 | Meaning: 0 | Mismatch: 0

Field	Manual/Ground Truth	LLM Extraction	Match
protocol	Smart-seq2	Smart-seq2	✓ Exact
sequencer	Illumina HiSeq 2000	Illumina HiSeq 2000	✓ Exact
tissue	developing ventral midbrain cells	developing ventral midbrain cells	✓ Exact
organism	Mus musculus	Mus musculus	✓ Exact
age	E12.5	E12.5	✓ Exact
genotype	C57BL/6 Heterozygous Lmx1a-EGFP	C57BL/6 Heterozygous Lmx1a-EGFP	✓ Exact
experiment_type	single-cell RNA-seq	single-cell RNA-seq	✓ Exact

GSM2320593 (Series: GSE87069)
Evaluable: 7 fields | Exact: 7 | Meaning: 0 | Mismatch: 0

Field	Manual/Ground Truth	LLM Extraction	Match
protocol	Smart-seq2	Smart-seq2	✓ Exact
sequencer	Illumina HiSeq 2000	Illumina HiSeq 2000	✓ Exact
tissue	developing ventral midbrain cells	developing ventral midbrain cells	✓ Exact
organism	Mus musculus	Mus musculus	✓ Exact
age	E12.5	E12.5	✓ Exact
genotype	C57BL/6 Heterozygous Lmx1a-EGFP	C57BL/6 Heterozygous Lmx1a-EGFP	✓ Exact
experiment_type	single-cell RNA-seq	single-cell RNA-seq	✓ Exact

GSM2320607 (Series: GSE87069)
Evaluable: 7 fields | Exact: 7 | Meaning: 0 | Mismatch: 0

Field	Manual/Ground Truth	LLM Extraction	Match
protocol	Smart-seq2	Smart-seq2	✓ Exact
sequencer	Illumina HiSeq 2000	Illumina HiSeq 2000	✓ Exact
tissue	developing ventral midbrain cells	developing ventral midbrain cells	✓ Exact
organism	Mus musculus	Mus musculus	✓ Exact
age	E12.5	E12.5	✓ Exact
genotype	C57BL/6 Heterozygous Lmx1a-EGFP	C57BL/6 Heterozygous Lmx1a-EGFP	✓ Exact
experiment_type	single-cell RNA-seq	single-cell RNA-seq	✓ Exact

GSM2320621 (Series: GSE87069)
Evaluable: 7 fields | Exact: 7 | Meaning: 0 | Mismatch: 0

Field	Manual/Ground Truth	LLM Extraction	Match
protocol	Smart-seq2	Smart-seq2	✓ Exact
sequencer	Illumina HiSeq 2000	Illumina HiSeq 2000	✓ Exact
tissue	developing ventral midbrain cells	developing ventral midbrain cells	✓ Exact
organism	Mus musculus	Mus musculus	✓ Exact
age	E12.5	E12.5	✓ Exact
genotype	C57BL/6 Heterozygous Lmx1a-EGFP	C57BL/6 Heterozygous Lmx1a-EGFP	✓ Exact
experiment_type	single-cell RNA-seq	single-cell RNA-seq	✓ Exact

GSM2320635 (Series: GSE87069)
Evaluable: 7 fields | Exact: 7 | Meaning: 0 | Mismatch: 0

Field	Manual/Ground Truth	LLM Extraction	Match
protocol	Smart-seq2	Smart-seq2	✓ Exact
sequencer	Illumina HiSeq 2000	Illumina HiSeq 2000	✓ Exact
tissue	developing ventral midbrain cells	developing ventral midbrain cells	✓ Exact
organism	Mus musculus	Mus musculus	✓ Exact
age	E12.5	E12.5	✓ Exact
genotype	C57BL/6 Heterozygous Lmx1a-EGFP	C57BL/6 Heterozygous Lmx1a-EGFP	✓ Exact
experiment_type	single-cell RNA-seq	single-cell RNA-seq	✓ Exact

GSM2320649 (Series: GSE87069)
Evaluable: 7 fields | Exact: 7 | Meaning: 0 | Mismatch: 0

Field	Manual/Ground Truth	LLM Extraction	Match
protocol	Smart-seq2	Smart-seq2	✓ Exact
sequencer	Illumina HiSeq 2000	Illumina HiSeq 2000	✓ Exact
tissue	developing ventral midbrain cells	developing ventral midbrain cells	✓ Exact
organism	Mus musculus	Mus musculus	✓ Exact
age	E12.5	E12.5	✓ Exact
genotype	C57BL/6 Heterozygous Lmx1a-EGFP	C57BL/6 Heterozygous Lmx1a-EGFP	✓ Exact
experiment_type	single-cell RNA-seq	single-cell RNA-seq	✓ Exact

GSM2320663 (Series: GSE87069)
Evaluable: 7 fields | Exact: 7 | Meaning: 0 | Mismatch: 0

Field	Manual/Ground Truth	LLM Extraction	Match
protocol	Smart-seq2	Smart-seq2	✓ Exact
sequencer	Illumina HiSeq 2000	Illumina HiSeq 2000	✓ Exact
tissue	developing ventral midbrain cells	developing ventral midbrain cells	✓ Exact
organism	Mus musculus	Mus musculus	✓ Exact
age	E12.5	E12.5	✓ Exact
genotype	C57BL/6 Heterozygous Lmx1a-EGFP	C57BL/6 Heterozygous Lmx1a-EGFP	✓ Exact
experiment_type	single-cell RNA-seq	single-cell RNA-seq	✓ Exact

GSM2320677 (Series: GSE87069)
Evaluable: 7 fields | Exact: 7 | Meaning: 0 | Mismatch: 0

Field	Manual/Ground Truth	LLM Extraction	Match
protocol	Smart-seq2	Smart-seq2	✓ Exact
sequencer	Illumina HiSeq 2000	Illumina HiSeq 2000	✓ Exact
tissue	developing ventral midbrain cells	developing ventral midbrain cells	✓ Exact
organism	Mus musculus	Mus musculus	✓ Exact
age	E13.5	E13.5	✓ Exact
genotype	C57BL/6 Heterozygous Lmx1a-EGFP	C57BL/6 Heterozygous Lmx1a-EGFP	✓ Exact
experiment_type	RNA-Seq	RNA-Seq	✓ Exact

GSM2320691 (Series: GSE87069)
Evaluable: 7 fields | Exact: 7 | Meaning: 0 | Mismatch: 0

Field	Manual/Ground Truth	LLM Extraction	Match
protocol	Smart-seq2	Smart-seq2	✓ Exact
sequencer	Illumina HiSeq 2000	Illumina HiSeq 2000	✓ Exact
tissue	developing ventral midbrain cells	developing ventral midbrain cells	✓ Exact
organism	Mus musculus	Mus musculus	✓ Exact
age	E13.5	E13.5	✓ Exact
genotype	C57BL/6 Heterozygous Lmx1a-EGFP	C57BL/6 Heterozygous Lmx1a-EGFP	✓ Exact
experiment_type	single-cell RNA-seq	single-cell RNA-seq	✓ Exact

GSM2320705 (Series: GSE87069)
Evaluable: 7 fields | Exact: 7 | Meaning: 0 | Mismatch: 0

Field	Manual/Ground Truth	LLM Extraction	Match
protocol	Smart-seq2	Smart-seq2	✓ Exact
sequencer	Illumina HiSeq 2000	Illumina HiSeq 2000	✓ Exact
tissue	developing ventral midbrain cells	developing ventral midbrain cells	✓ Exact
organism	Mus musculus	Mus musculus	✓ Exact
age	E13.5	E13.5	✓ Exact
genotype	C57BL/6 Heterozygous Lmx1a-EGFP	C57BL/6 Heterozygous Lmx1a-EGFP	✓ Exact
experiment_type	single-cell RNA-seq	single-cell RNA-seq	✓ Exact

GSM2320719 (Series: GSE87069)
Evaluable: 7 fields | Exact: 7 | Meaning: 0 | Mismatch: 0

Field	Manual/Ground Truth	LLM Extraction	Match
protocol	Smart-seq2	Smart-seq2	✓ Exact
sequencer	Illumina HiSeq 2000	Illumina HiSeq 2000	✓ Exact
tissue	developing ventral midbrain cells	developing ventral midbrain cells	✓ Exact
organism	Mus musculus	Mus musculus	✓ Exact
age	E13.5	E13.5	✓ Exact
genotype	C57BL/6 Heterozygous Lmx1a-EGFP	C57BL/6 Heterozygous Lmx1a-EGFP	✓ Exact
experiment_type	single-cell RNA-seq	single-cell RNA-seq	✓ Exact

GSM2320733 (Series: GSE87069)
Evaluable: 7 fields | Exact: 7 | Meaning: 0 | Mismatch: 0

Field	Manual/Ground Truth	LLM Extraction	Match
protocol	Smart-seq2	Smart-seq2	✓ Exact
sequencer	Illumina HiSeq 2000	Illumina HiSeq 2000	✓ Exact
tissue	developing ventral midbrain cells	developing ventral midbrain cells	✓ Exact
organism	Mus musculus	Mus musculus	✓ Exact
age	E13.5	E13.5	✓ Exact
genotype	C57BL/6 Heterozygous Lmx1a-EGFP	C57BL/6 Heterozygous Lmx1a-EGFP	✓ Exact
experiment_type	single-cell RNA-seq	single-cell RNA-seq	✓ Exact

GSM2320747 (Series: GSE87069)
Evaluable: 7 fields | Exact: 7 | Meaning: 0 | Mismatch: 0

Field	Manual/Ground Truth	LLM Extraction	Match
protocol	Smart-seq2	Smart-seq2	✓ Exact
sequencer	Illumina HiSeq 2000	Illumina HiSeq 2000	✓ Exact
tissue	developing ventral midbrain cells	developing ventral midbrain cells	✓ Exact
organism	Mus musculus	Mus musculus	✓ Exact
age	E13.5	E13.5	✓ Exact
genotype	C57BL/6 Heterozygous Lmx1a-EGFP	C57BL/6 Heterozygous Lmx1a-EGFP	✓ Exact
experiment_type	single-cell RNA-seq	single-cell RNA-seq	✓ Exact

GSM2320761 (Series: GSE87069)
Evaluable: 7 fields | Exact: 7 | Meaning: 0 | Mismatch: 0

Field	Manual/Ground Truth	LLM Extraction	Match
protocol	Smart-seq2	Smart-seq2	✓ Exact
sequencer	Illumina HiSeq 2000	Illumina HiSeq 2000	✓ Exact
tissue	developing ventral midbrain cells	developing ventral midbrain cells	✓ Exact
organism	Mus musculus	Mus musculus	✓ Exact
age	E13.5	E13.5	✓ Exact
genotype	C57BL/6 Heterozygous Lmx1a-EGFP	C57BL/6 Heterozygous Lmx1a-EGFP	✓ Exact
experiment_type	single-cell RNA-seq	single-cell RNA-seq	✓ Exact

GSM2320775 (Series: GSE87069)
Evaluable: 7 fields | Exact: 7 | Meaning: 0 | Mismatch: 0

Field	Manual/Ground Truth	LLM Extraction	Match
protocol	Smart-seq2	Smart-seq2	✓ Exact
sequencer	Illumina HiSeq 2000	Illumina HiSeq 2000	✓ Exact
tissue	developing ventral midbrain cells	developing ventral midbrain cells	✓ Exact
organism	Mus musculus	Mus musculus	✓ Exact
age	E13.5	E13.5	✓ Exact
genotype	C57BL/6 Heterozygous Lmx1a-EGFP	C57BL/6 Heterozygous Lmx1a-EGFP	✓ Exact
experiment_type	RNA-Seq	RNA-Seq	✓ Exact

GSM2320789 (Series: GSE87069)
Evaluable: 7 fields | Exact: 7 | Meaning: 0 | Mismatch: 0

Field	Manual/Ground Truth	LLM Extraction	Match
protocol	Smart-seq2	Smart-seq2	✓ Exact
sequencer	Illumina HiSeq 2000	Illumina HiSeq 2000	✓ Exact
tissue	developing ventral midbrain cells	developing ventral midbrain cells	✓ Exact
organism	Mus musculus	Mus musculus	✓ Exact
age	E13.5	E13.5	✓ Exact
genotype	C57BL/6 Heterozygous Lmx1a-EGFP	C57BL/6 Heterozygous Lmx1a-EGFP	✓ Exact
experiment_type	single-cell RNA-seq	single-cell RNA-seq	✓ Exact

GSM2320803 (Series: GSE87069)
Evaluable: 7 fields | Exact: 7 | Meaning: 0 | Mismatch: 0

Field	Manual/Ground Truth	LLM Extraction	Match
protocol	Smart-seq2	Smart-seq2	✓ Exact
sequencer	Illumina HiSeq 2000	Illumina HiSeq 2000	✓ Exact
tissue	developing ventral midbrain cells	developing ventral midbrain cells	✓ Exact
organism	Mus musculus	Mus musculus	✓ Exact
age	E13.5	E13.5	✓ Exact
genotype	C57BL/6 Heterozygous Lmx1a-EGFP	C57BL/6 Heterozygous Lmx1a-EGFP	✓ Exact
experiment_type	single-cell RNA-seq	single-cell RNA-seq	✓ Exact

GSM2320817 (Series: GSE87069)
Evaluable: 7 fields | Exact: 7 | Meaning: 0 | Mismatch: 0

Field	Manual/Ground Truth	LLM Extraction	Match
protocol	Smart-seq2	Smart-seq2	✓ Exact
sequencer	Illumina HiSeq 2000	Illumina HiSeq 2000	✓ Exact
tissue	developing ventral midbrain cells	developing ventral midbrain cells	✓ Exact
organism	Mus musculus	Mus musculus	✓ Exact
age	E11.5	E11.5	✓ Exact
genotype	C57BL/6 Heterozygous Lmx1a-EGFP	C57BL/6 Heterozygous Lmx1a-EGFP	✓ Exact
experiment_type	RNA-seq	RNA-seq	✓ Exact

GSM2320831 (Series: GSE87069)
Evaluable: 7 fields | Exact: 7 | Meaning: 0 | Mismatch: 0

Field	Manual/Ground Truth	LLM Extraction	Match
protocol	Smart-seq2	Smart-seq2	✓ Exact
sequencer	Illumina HiSeq 2000	Illumina HiSeq 2000	✓ Exact
tissue	developing ventral midbrain cells	developing ventral midbrain cells	✓ Exact
organism	Mus musculus	Mus musculus	✓ Exact
age	E11.5	E11.5	✓ Exact
genotype	C57BL/6 Heterozygous Lmx1a-EGFP	C57BL/6 Heterozygous Lmx1a-EGFP	✓ Exact
experiment_type	single-cell RNA-seq	single-cell RNA-seq	✓ Exact

GSM2320845 (Series: GSE87069)
Evaluable: 7 fields | Exact: 7 | Meaning: 0 | Mismatch: 0

Field	Manual/Ground Truth	LLM Extraction	Match
protocol	Smart-seq2	Smart-seq2	✓ Exact
sequencer	Illumina HiSeq 2000	Illumina HiSeq 2000	✓ Exact
tissue	developing ventral midbrain cells	developing ventral midbrain cells	✓ Exact
organism	Mus musculus	Mus musculus	✓ Exact
age	E11.5	E11.5	✓ Exact
genotype	C57BL/6 Heterozygous Lmx1a-EGFP	C57BL/6 Heterozygous Lmx1a-EGFP	✓ Exact
experiment_type	single-cell RNA-seq	single-cell RNA-seq	✓ Exact

GSM2320859 (Series: GSE87069)
Evaluable: 7 fields | Exact: 7 | Meaning: 0 | Mismatch: 0

Field	Manual/Ground Truth	LLM Extraction	Match
protocol	Smart-seq2	Smart-seq2	✓ Exact
sequencer	Illumina HiSeq 2000	Illumina HiSeq 2000	✓ Exact
tissue	developing ventral midbrain cells	developing ventral midbrain cells	✓ Exact
organism	Mus musculus	Mus musculus	✓ Exact
age	E11.5	E11.5	✓ Exact
genotype	C57BL/6 Heterozygous Lmx1a-EGFP	C57BL/6 Heterozygous Lmx1a-EGFP	✓ Exact
experiment_type	RNA-Seq	RNA-Seq	✓ Exact

GSM2320873 (Series: GSE87069)
Evaluable: 7 fields | Exact: 7 | Meaning: 0 | Mismatch: 0

Field	Manual/Ground Truth	LLM Extraction	Match
protocol	Smart-seq2	Smart-seq2	✓ Exact
sequencer	Illumina HiSeq 2000	Illumina HiSeq 2000	✓ Exact
tissue	developing ventral midbrain cells	developing ventral midbrain cells	✓ Exact
organism	Mus musculus	Mus musculus	✓ Exact
age	E11.5	E11.5	✓ Exact
genotype	C57BL/6 Heterozygous Lmx1a-EGFP	C57BL/6 Heterozygous Lmx1a-EGFP	✓ Exact
experiment_type	RNA-Seq	RNA-Seq	✓ Exact

GSM2320887 (Series: GSE87069)
Evaluable: 7 fields | Exact: 7 | Meaning: 0 | Mismatch: 0

Field	Manual/Ground Truth	LLM Extraction	Match
protocol	Smart-seq2	Smart-seq2	✓ Exact
sequencer	Illumina HiSeq 2000	Illumina HiSeq 2000	✓ Exact
tissue	developing ventral midbrain cells	developing ventral midbrain cells	✓ Exact
organism	Mus musculus	Mus musculus	✓ Exact
age	E10.5	E10.5	✓ Exact
genotype	C57BL/6 Heterozygous Lmx1a-EGFP	C57BL/6 Heterozygous Lmx1a-EGFP	✓ Exact
experiment_type	RNA-seq	RNA-seq	✓ Exact

GSM2320901 (Series: GSE87069)
Evaluable: 7 fields | Exact: 7 | Meaning: 0 | Mismatch: 0

Field	Manual/Ground Truth	LLM Extraction	Match
protocol	Smart-seq2	Smart-seq2	✓ Exact
sequencer	Illumina HiSeq 2000	Illumina HiSeq 2000	✓ Exact
tissue	developing ventral midbrain cells	developing ventral midbrain cells	✓ Exact
organism	Mus musculus	Mus musculus	✓ Exact
age	E13.5	E13.5	✓ Exact
genotype	C57BL/6 Heterozygous Lmx1a-EGFP	C57BL/6 Heterozygous Lmx1a-EGFP	✓ Exact
experiment_type	RNA-seq	RNA-seq	✓ Exact
