# Supplementary figures and images for: Celline: a flexible tool for one-step retrieval and integrative analysis of public single-cell RNA sequencing data
Source: Front Bioinform. 2025 Dec 11;5:1684227. doi: 10.3389/fbinf.2025.1684227 (PMC12738925; doi:10.3389/fbinf.2025.1684227)

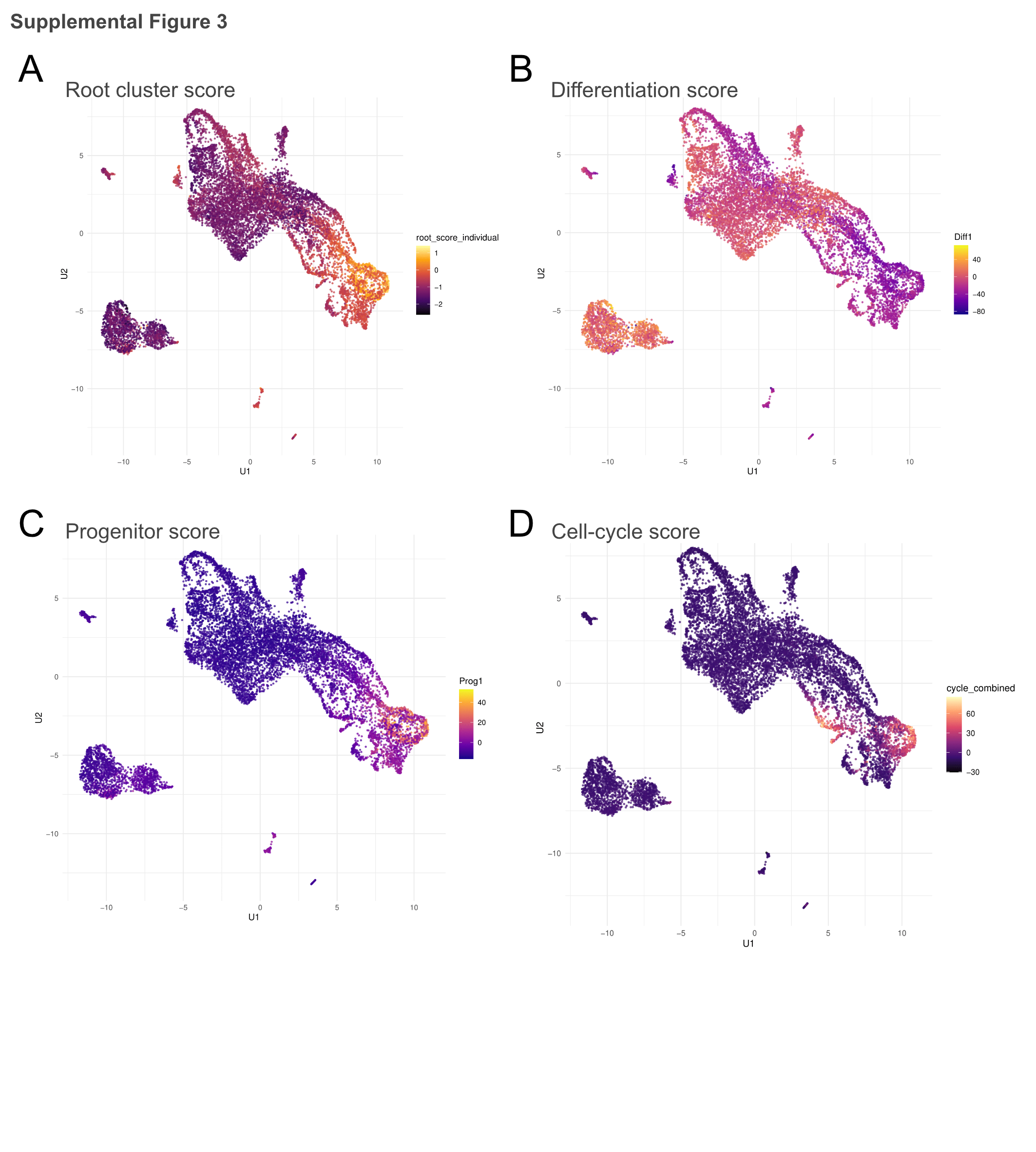

Supplement: Supplementary file 2 [file Image3.jpeg]

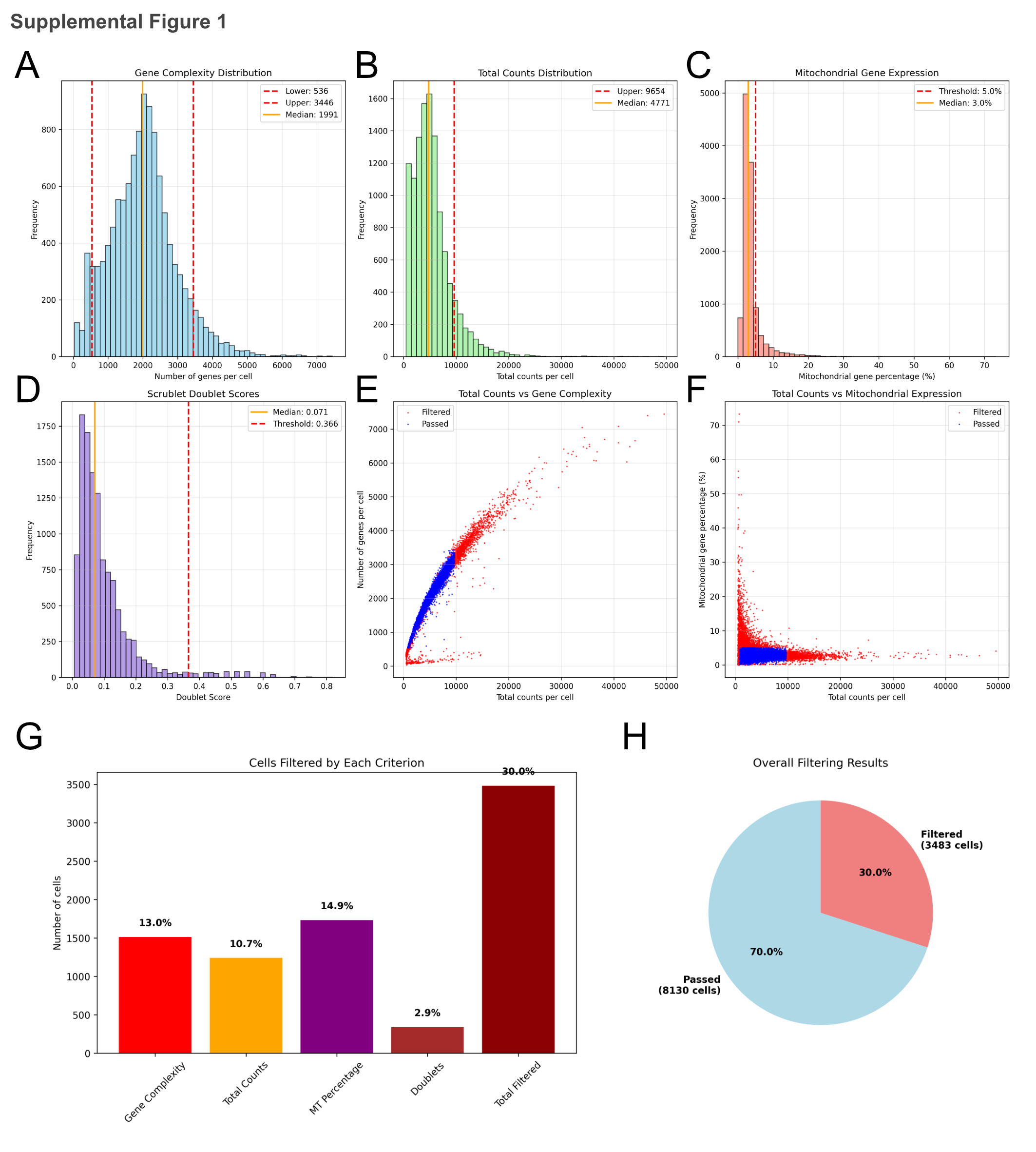

Supplement: Supplementary file 3 [file Image1.jpeg]

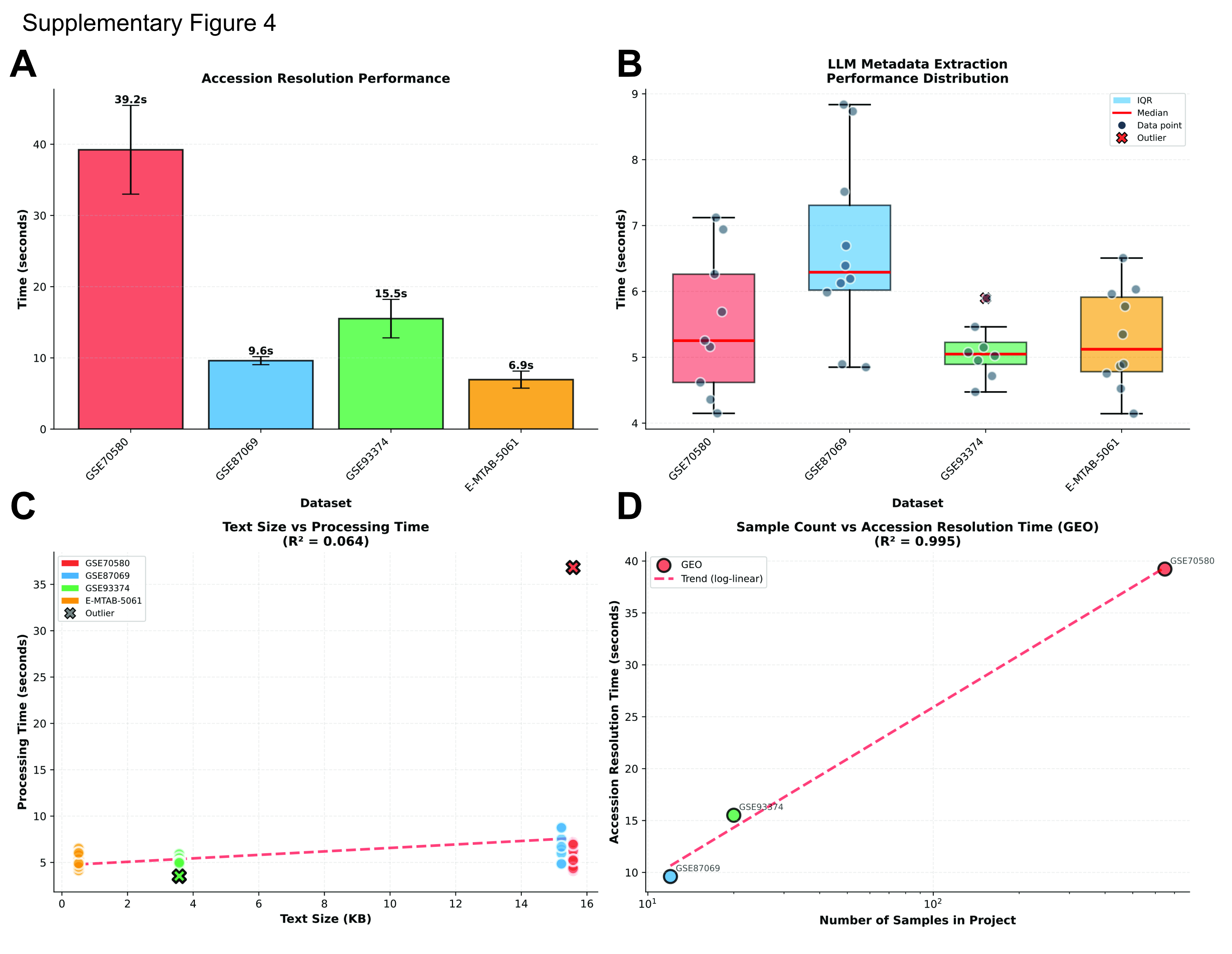

Supplement: Supplementary file 4 [file Image4.jpeg]

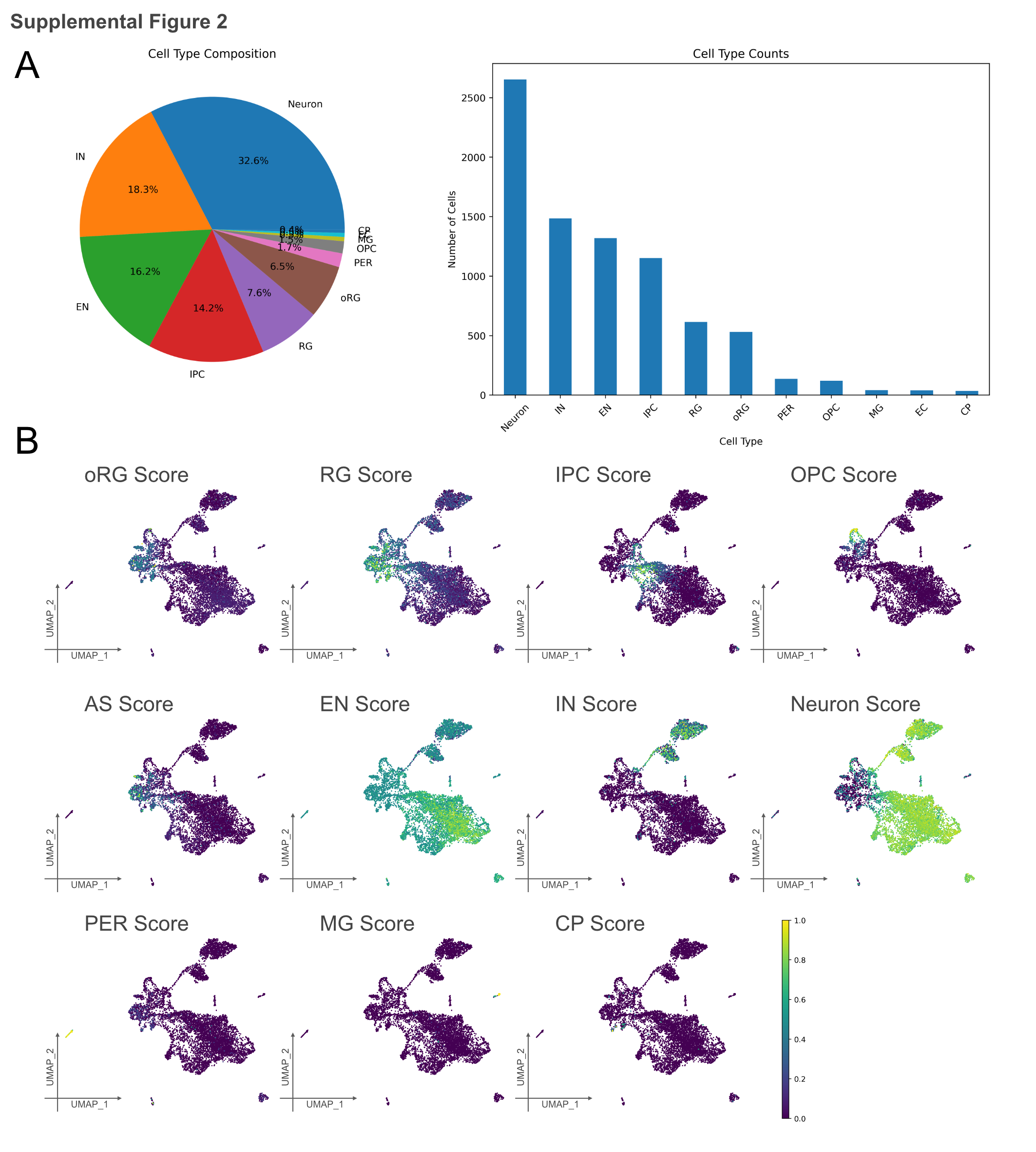

Supplement: Supplementary file 5 [file Image2.jpeg]

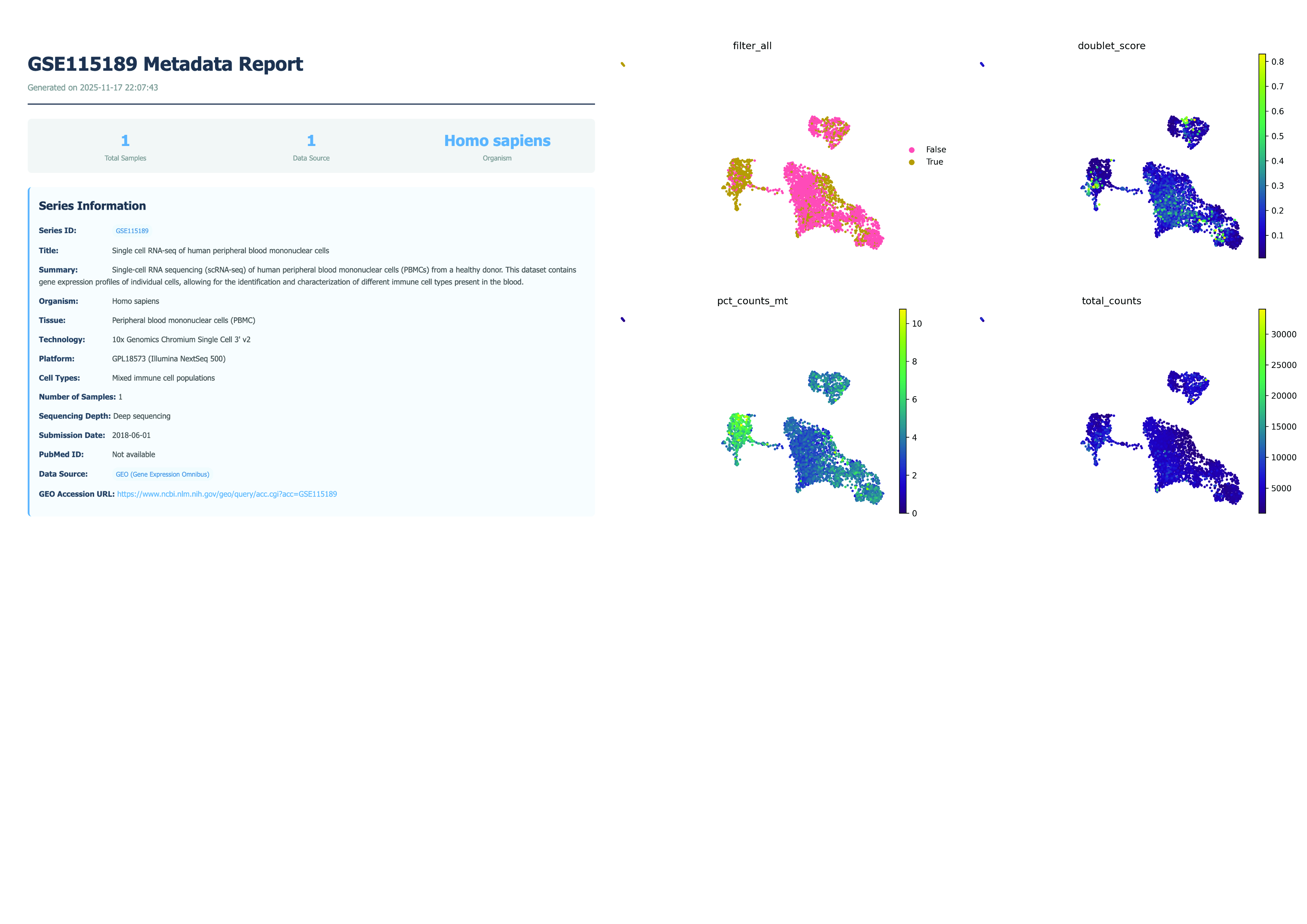

Supplement: Supplementary file 6 [file Image5.jpeg]
