# Supplementary material for: Celline: a flexible tool for one-step retrieval and integrative analysis of public single-cell RNA sequencing data
Source: Front Bioinform. 2025 Dec 11;5:1684227. doi: 10.3389/fbinf.2025.1684227 (PMC12738925; doi:10.3389/fbinf.2025.1684227)
Supplement: Supplementary file 7 [file DataSheet1.pdf]

<table>
<tr><th>Step</th><th>Command</th><th>Description</th><th>Key Parameters</th><th>Output</th></tr>
<tr><td>1.
Initialize</td><td>celline init</td><td>Initialize new project and validate system dependencies
(R, Cell Ranger, SRA Toolkit)</td><td>Interactive setup
(project name, R path)</td><td>setting.toml
samples.toml</td></tr>
<tr><td>2.
Add Samples</td><td>celline run add
<sample_id> ...</td><td>Register samples from public databases
(GEO, SRA, CNCB) or local data</td><td>sample_ids (positional)
GSE/GSM/SRR/CRA IDs</td><td>Updated
samples.toml</td></tr>
<tr><td>3.
Download</td><td>celline run
download</td><td>Download raw sequencing data
(FASTQ/BAM files) for all samples</td><td>--nthread <N>
--filetype <fastq|bam></td><td>FASTQ/BAM files
in resources/</td></tr>
<tr><td>4.
Count</td><td>celline run
count</td><td>Gene expression quantification
using Cell Ranger or STARsolo</td><td>--nthread <N>
--transcriptome <ref>
--counter <cellranger|starsolo></td><td>Count matrices
filtered_feature_bc
_matrix.h5</td></tr>
<tr><td>5.
Preprocess</td><td>celline run
preprocess</td><td>QC filtering, doublet detection,
normalization, PCA, UMAP</td><td>--mt-pct-threshold <N>
(default: 5.0)
--n-mad <N>
(default: 2.5)
--target-celltype <type></td><td>QC plots
UMAP plots
cell_info.tsv</td></tr>
<tr><td>6.
Cell Type
Prediction</td><td>celline run
predict_celltype</td><td>Automated cell type annotation
using markers or scPred</td><td>--mode <canonical|reference>
(default: canonical)
--marker-path <file>
--species <name></td><td>celltype_predicted.tsv
Visualization plots</td></tr>
<tr><td>7.
Integrate</td><td>celline run
integrate</td><td>Batch effect correction and
data integration across samples</td><td>--method <harmony|scvi>
(default: harmony)
--n-pcs <N>
--sample <id,id,...></td><td>Integrated h5ad
Integration plots</td></tr>
</table>

8. Batch Correction	celline run batch_cor	Alternative batch correction methods for technical variation	--method <type>	Corrected expression matrix
9. Create Seurat	celline run create_seurat	Create Seurat RDS objects for R-based downstream analysis	--useqc-matrix (use QC-filtered matrix)	seurat.rds (per sample)
10. Export Metareport	celline run export_metareport	Generate comprehensive HTML metadata report with statistics	--output <filename> (default: metadata_report.html) --use-ai (AI analysis)	HTML metadata report with charts
11. Reduce	celline run reduce	Clean up intermediate files to save disk space	Automatic (keeps essential files)	Reduced storage size
12. Info	celline run info	Display current project status and analysis progress	None	Project status report
